# Engineered Biomimetic Fibrillar Fibronectin Matrices Regulate Cell Adhesion Initiation, Migration, and Proliferation via *α*5*β*1 Integrin and Syndecan‐4 Crosstalk

**DOI:** 10.1002/advs.202300812

**Published:** 2023-06-25

**Authors:** Seungkuk Ahn, Upnishad Sharma, Krishna Chaitanya Kasuba, Nico Strohmeyer, Daniel J. Müller

**Affiliations:** ^1^ Department of Biosystems Science and Engineering Eidgenössische Technische Hochschule (ETH) Zurich Basel 4058 Switzerland

**Keywords:** 3D fibrillar fibronectin, adhesion initiation, extracellular matrix, integrin, spatiotemporal cell dynamics, syndecan

## Abstract

Cells regulate adhesion to the fibrillar extracellular matrix (ECM) of which fibronectin is an essential component. However, most studies characterize cell adhesion to globular fibronectin substrates at time scales long after cells polarize and migrate. To overcome this limitation, a simple and scalable method to engineer biomimetic 3D fibrillar fibronectin matrices is introduced and how they are sensed by fibroblasts from the onset of attachment is characterized. Compared to globular fibronectin substrates, fibroblasts accelerate adhesion initiation and strengthening within seconds to fibrillar fibronectin matrices via *α*5*β*1 integrin and syndecan‐4. This regulation, which additionally accelerates on stiffened fibrillar matrices, involves actin polymerization, actomyosin contraction, and the cytoplasmic proteins paxillin, focal adhesion kinase, and phosphoinositide 3‐kinase. Furthermore, this immediate sensing and adhesion of fibroblast to fibrillar fibronectin guides migration speed, persistency, and proliferation range from hours to weeks. The findings highlight that fibrillar fibronectin matrices, compared to widely‐used globular fibronectin, trigger short‐ and long‐term cell decisions very differently and urge the use of such matrices to better understand in vivo interactions of cells and ECMs. The engineered fibronectin matrices, which can be printed onto non‐biological surfaces without loss of function, open avenues for various cell biological, tissue engineering and medical applications.

## Graphical abstract

Biomimetic fibrillar fibronectin matrices are engineered using 3D‐printed microgrids and simple solution shearing. Attaching to fibrillar fibronectin matrices, fibroblasts strengthen adhesion initiation within seconds and enhance long‐term migration and proliferation by employing *β*1 integrin and syndecan‐4. This rapid and continual fibrillar mechanosensing explains how fibrillar extracellular matrices modulate cell decisions in native tissues.

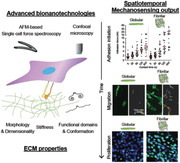

## 1 Introduction

Tissue‐specific biochemical compositions and biophysical properties of 3D fibrillar extracellular matrices (ECM) modulate the cellular response and hence play crucial roles in physiology and pathology.^[^ ^1^ ^]^ Fibronectin (FN) is a structural and biochemical component of the ECM that is indispensable for development, homeostasis, and wound healing.^[^ ^2^ ^]^ FN consists of three different domains FNI, FNII, and FNIII, which are arranged like pearls on a string and harbor several interaction sites for cell adhesion molecules including integrins and syndecans. Integrin binding and subsequent actomyosin‐driven contractile forces unfold the secreted globular FN and induces the assembly of FN fibrils, which eventually leads to the formation of a complex insoluble FN fibrillar network.^[^ ^3^ ^]^ The network exposes FN binding sites for other cell adhesion receptors, growth factors, and ECM proteins. FN thus serves as a scaffold to assemble complex 3D multifunctional ECMs to maintain tissue structure and function.^[^ ^2^ ^]^ Pathologies including chronic inflammation, fibrosis, and cancer progression are related to abnormal FN deposition and stiffening, which highlights the importance of the structural and mechanical properties of FN fibrillar networks in tissue.^[^ ^4^ ^]^

Cells employ heterodimeric integrins to sense and adhere to complex ECM networks.^[^ ^5^ ^]^ In mammals, 18 *α* and 8 *β* subunits form 24 different integrins, many of which being co‐expressed in cells. Most FN‐binding integrins, in particular *α*5*β*1 and *α*V*β*3 integrins, bind to the RGD tripeptide in the tenth FNIII repeat.^[^ ^6^ ^]^ Additionally, *α*5*β*1 integrin binds to the PHSRN synergy site located in the ninth FNIII repeat, which is required to establish catch‐bonds for mechanotransduction.^[^ ^7^ ^]^ Upon ligand binding, integrins cluster and recruit hundreds of intracellular adaptors and signaling proteins to their cytoplasmic domain to assemble macromolecular cell adhesion sites.^[^ ^8^ ^]^ Integrins commonly crosstalk with other transmembrane receptors, including syndecans to sense and respond to the complex information provided by the ECM.^[^ ^9^ ^]^ Syndecans are a family of four transmembrane proteoglycans, which bind to a variety of ligands, including the heparin‐binding domains in the twelfth–fourteenth FNIII repeats.^[^ ^2^ ^]^ Syndecan‐binding to FN triggers downstream signaling and regulates cell adhesion, migration, and proliferation. In addition, syndecans cooperate with integrins to promote F‐actin dynamics, mechanotransduction,^[^ ^9^, ^10^ ^]^ and ECM assembly.^[^ ^6^, ^11^ ^]^ The expression level of syndecans in cancer cells has been associated with tumor size, invasiveness, and metastatic capacity.^[^ ^12^ ^]^ However, to date the investigation of the crosstalk between syndecan and integrin focuses mainly on long‐term (hours to days) adhesion, migration, and actin regulation. Whether, how, and which syndecans crosstalk with integrins during adhesion initiation to determine adhesion dynamics, adhesome composition, adhesion strengthening, and how this crosstalk translates into cellular processes at the long‐term thus remains largely elusive.

To investigate molecular pathways of cell adhesion with regards to integrin‐FN interaction, simplified FN models such as peptidomimetics (e.g., RGD peptides),^[^ ^13^ ^]^ FN fragments,^[^ ^7^, ^14^ ^]^ or globular FN coating^[^ ^15^ ^]^ are commonly used. Such simplified models may not fully embody the various biochemical and biophysical properties of the fibrillar FN matrix in native tissues. Additionally, cell adhesion has mostly been characterized at longer time scales (e.g., after 60–90 min).^[^ ^1^, ^13^, ^16^ ^]^ However, to better understand spatiotemporal cell dynamics, it is essential to characterize how cells sense and respond to fibrillar FN matrices starting from the onset of adhesion.

Previous in vitro studies have unfolded globular FN and initiated the self‐assembly of FN fibrils by extracting fibers from FN solution,^[^ ^17^ ^]^ applying surface charges,^[^ ^18^ ^]^ using denaturants,^[^ ^19^ ^]^ or employing protein‐surface interactions.^[^ ^20^ ^]^ However, these attempts possess limitations such as 2D substrates instead of 3D matrices, manual fiber deposition, poor transparency, low throughput, hard to scale up, low porosity, or poor cell infiltration. A recently introduced approach, aiming to overcome such shortcomings, induces the formation of 3D fibrillar FN matrices under hydrodynamic shear force between an electrospun support, fibronectin solution, and air.^[^ ^21^ ^]^ Yet, the cumbersome, costly, and non‐standard electrospinning set‐up limits the wide adaptability and applicability of this method.

Here, we introduce a cost‐efficient and adaptable method, which uses 3D‐printed porous microgrids to grow 3D fibrillar FN matrices that expose nano‐ and micro‐fibrillar structures, stiffnesses and cryptic binding sites similar to fibrillar FN matrices produced in vitro. By systematically comparing how fibroblasts sense the biomimetic 3D fibrillar matrices differently compared to commonly used 2D globular FN substrates, we reveal that during adhesion initiation *α*5*β*1 integrins crosstalk with syndecan‐4 to sense the fibrillarity and stiffness of FN matrices. This rapid sensing of FN fibrillarity and mechanical cues, which is absent on globular FN substrates, is transduced by multiple intracellular pathways to strengthen fibroblast adhesion within seconds. We show that fibroblasts employ this rapid sensing mechanism to guide long‐time cellular processes lasting from hours to weeks such as cell migration, persistency, and proliferation. Importantly, the 3D fibrillar FN matrices can be printed onto inorganic surfaces and thus open an avenue for the biofunctionalization of various materials.

## 2 Results and Discussion

### 2.1 Engineering Fibrillar FN Matrices Using 3D‐Printed Grids

**Figure 1:**
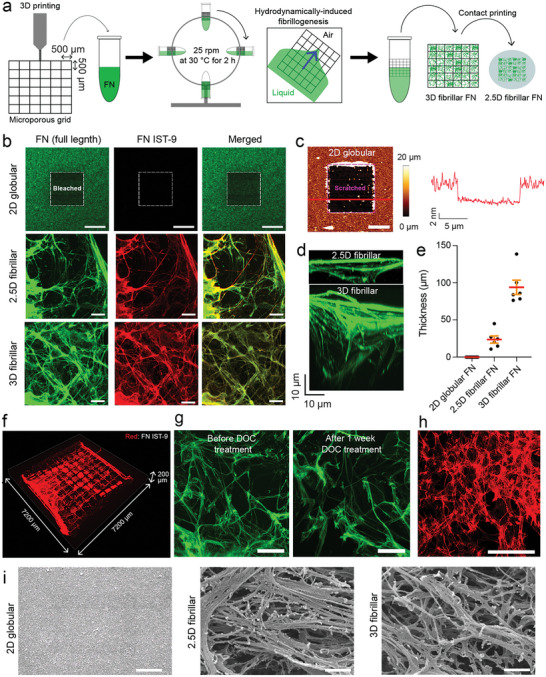
Engineering 2.5D and 3D fibrillar fibronectin (FN) matrices. a) Schematic illustration of engineering 3D fibrillar FN matrices and 2.5D fibrillar FN matrices. First, a microporous grid (500 × 500 µm^2^ square pores, 200 µm thickness and a size of ≈6 × 6 mm^2^) made from an ABS‐like resin is 3D printed. The grid is placed in an Eppendorf tube filled with full‐length FN in PBS. The tube is then rotated (25 rpm) at 30 °C for 2 h to apply hydrodynamic shear force between the microgrid, FN solution, and air. After rotation, the grid embedded with a 3D fibrillar FN matrix can be removed from the Eppendorf tube and used. Contacting printing 3D fibrillar FN matrices onto glass covers the inorganic substrate with 2.5D fibrillar FN matrices. b) Representative fluorescence images of 2D globular FN substrates, 2.5D fibrillar FN matrices, and 3D fibrillar FN matrices. Fluorescence images show full‐length FN (green), unfolded FN (FN IST‐9, red), and both images merged. Dashed boxes in 2D globular FN substrates indicate the bleached area to distinguish fluorescent signal from background. Scale bars, 20 µm. c) AFM topography of 2D globular FN substrates scratched in the middle square area (purple dashed square). The height profile (red line) is generated along the red line in the topography. Scale bar, 5 µm. d) *XZ* projection confocal images of fibrillar FN matrices (green, full‐length FN antibody). Scale bar, 10 µm. e) Thickness analysis of globular FN substrates and fibrillar FN matrices. The mean thickness is 6.50 ± 0.31 nm for 2D globular FN substrates, 23.82 ± 4.93 µm for 2.5D fibrillar FN matrices, and 94.12 ± 9.59 µm for 3D fibrillar FN matrices. Dots represent the number of samples analyzed. Red bars indicate the mean and orange bars the standard error of the mean (s.e.). f) 3D reconstruction of confocal images showing a large‐scale coverage of fibrillar FN matrices across the 3D microporous grid. g) Engineered 3D fibrillar FN matrices are insoluble in DOC. Engineered 3D fibrillar FN matrices were treated with deoxycholate (DOC) solution (1% w/v) in PBS for a week. Confocal images of fluorescently stained (full‐length FN antibody, green) FN matrices were recorded before and 1 week after DOC treatment. Scale bars, 50 µm. h) Fluorescence image of fibrillar FN matrices (FN IST‐9, red) deposited by fibroblasts in vitro. Scale bar, 50 µm. i) Representative scanning electron microscopy (SEM) images of 2D globular FN substrates, 2.5D fibrillar FN matrices, and 3D fibrillar matrices. Scale bars, 1 µm.

To engineer 3D fibrillar FN matrices, we used full‐length FN as a model ECM component. Outsourcing from a commercial 3D printing service (Protolabs), we 3D‐printed acrylonitrile butadiene styrene (ABS)‐like resin (MicroFine, Protolabs) into 200 µm thick scaffolding grids having 500 × 500 µm^2^ square pores, which extend over 6 × 6 mm^2^ in size (**Figure** **1**a). While simply rotating a 3D‐printed grid together with FN solution at 25 rpm for 2 h at 30 °C, we applied shear forces to unfold FN at three interfaces of air, grid, and aqueous FN solution.^[^ ^21^ ^]^ This process embedded the grid within a 3D fibrillar FN matrix having a thickness of 94 ± 9 µm (mean ± s.e.; Figure 1a–e). The rapid prototyping, reproducibility, and ease of preparation allowed to produce fibrillar FN matrices across the entire 3D printed grids, shows the simple scalability of the method (Figure 1f). Next, we transferred the 3D fibrillar FN matrices from the grid onto inorganic surfaces. Thereto, we contact‐printed^[^ ^22^ ^]^ the 3D fibrillar FN matrices onto a glass‐bottomed Petri dish, which produced 2.5D printed fibrillar FN matrices with a reduced thickness of 23 ± 4 µm (Figure 1a–e).

To directly compare fibrillar FN matrices with conventionally used non‐fibrillar FN substrates, we adsorbed globular FN onto glass‐bottomed Petri dishes, where it formed a 6.50 ± 0.31 nm thick layer^[^ ^23^ ^]^ (Figure 1b–e). All FN substrates showed immunofluorescent signals from antibodies binding full‐length FN, while only 3D and 2.5D fibrillar matrices showed immunofluorescent signals from FN IST‐9 antibodies recognizing unfolded FN^[^ ^24^ ^]^ (Figure 1b), as usually observed for FN fibrils deposited by cells. The engineered 3D fibrillar FN matrices remained insoluble after 1 week of deoxycholate (DOC) treatment (Figure 1g), which is known to dissolve only globular FN, but not fibrillar FN.^[^ ^21a^ ^]^ Further, we confirmed morphological similarities between engineered 3D and 2.5D fibrillar FN matrices and fibrillar FN matrices deposited by fibroblasts on Petri dishes by confocal microscopy using the FN IST‐9 antibody (Figure 1h). SEM images showed that the 3D fibrillar FN matrices and 2.5D printed fibrillar FN matrices are composed of micro‐ and nanofibrils that mimic a hierarchical fibrillar architecture that resembles native ECM microenvironments (Figure 1i).^[^ ^25^ ^]^ The results do not show any changes in the morphology or density of the fibrillar FN between 2.5D and 3D fibrillar matrices. Hence, we conclude that the thickness reduction of 2.5D compared to 3D fibrillar FN matrices occurs solely due to the contact printing technique.^[^ ^22^, ^26^ ^]^ While the FN fibers of the 3D fibrillar FN matrices in contact with the glass remain on the glass support upon removal of the 3D‐printed grid, FN fibrils that were not in contact with the glass remained within the grid. Importantly, the findings show the similarity between the engineered fibrillar FN matrices and fibrillar FN matrices natively grown in tissues.

Altogether, we introduced a relatively easy, fast, and scalable method to produce biomimetic 3D fibrillar FN matrices. The production of the biomimetic FN matrices requires only standard laboratory equipment, making it easy to reproduce and widely applicable. Importantly for diverse applications, the 3D FN matrices can be printed onto inorganic surfaces for their functionalization. However, we did not investigate the densities of ligands (e.g., RGD, PHSRN, or syndecan binding sites) of fibrillar FN matrices and globular FN substrates, which may differ between fibrillar matrices and globular substrates.^[^ ^27^ ^]^

### 2.2 Fibroblasts Accelerate Adhesion Initiation and Strengthening to Fibrillar FN Matrices via *α*5*β*1 Integrin

The contact to FN influences the short‐ and long‐term behavior of fibroblasts.^[^ ^28^ ^]^ Hence, we aim to understand whether and how fibroblasts sense and respond to the fibrillarity of the FN matrices upon establishing adhesion. To this end, we quantified the adhesion force of fibroblasts expressing distinct sets of FN‐binding integrins to globular and fibrillar FN matrices using atomic force microscopy (AFM)‐based single‐cell force spectroscopy (SCFS)^[^ ^29^ ^]^ (Figure S1, Supporting Information). We used pan‐integrin null (pKO) fibroblast lines lacking integrin expression and pKO fibroblasts reconstituted with *β*1‐class integrins (pKO‐*β*1, expressing FN‐binding *α*5*β*1 integrin), *α*V‐class integrins (pKO‐*α*V, expressing FN‐binding *α*V*β*3 integrin), or both FN‐binding integrin classes (pKO‐*α*V/*β*1).^[^ ^15b^ ^]^ For SCFS, we attached single fibroblasts to concanavalin A (ConA)‐coated cantilevers and brought them into contact with fibrillar FN matrices or globular FN substrates to initiate and strengthen adhesion for contact times of 5, 20, 50, 120, or 240 s. At the given contact time, we retracted the fibroblasts from the FN and quantified their adhesion force as the maximum downward deflection of the cantilever. We then determined the adhesion strengthening of each fibroblast line as the slope of their adhesion force increasing over contact time (Figure S2, Supporting Information).

**Figure 2:**
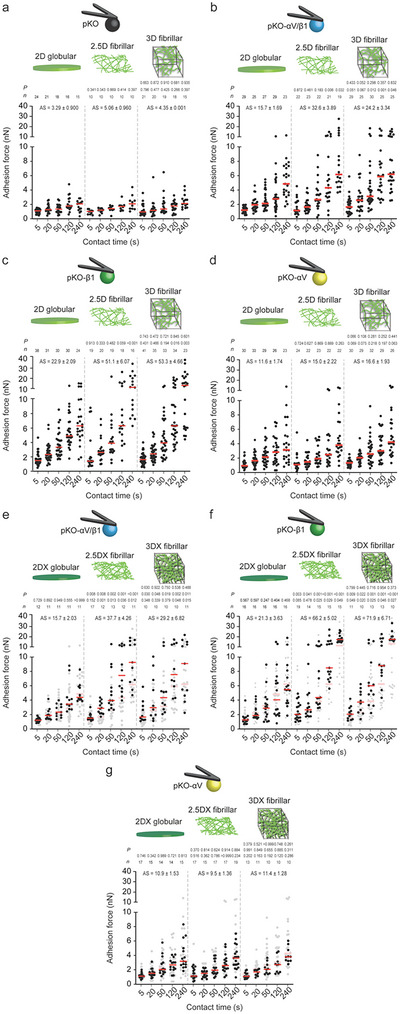
Fibroblasts employ *α*5*β*1 integrin to differentiate between globular FN substrates, fibrillar FN matrices, and stiffened fibrillar FN matrices to strengthen adhesion faster. a–d) Adhesion forces of pan‐integrin‐null (pKO) fibroblasts expressing no FN‐binding integrins (a), pKO fibroblasts expressing *α*5*β*1 and *α*V*β*3 integrins (pKO‐*α*V/*β*1) (b), pKO fibroblasts expressing *α*5*β*1 integrin (pKO‐*β*1) (c) or pKO fibroblasts expressing *α*V*β*3 integrin (pKO‐*α*V) to 2D globular FN substrates, 2.5D fibrillar FN matrices, and 3D fibrillar FN matrices at given contact times (d). Dots represent adhesion forces of individual fibroblasts and red bars the median. *n* indicates the number of fibroblasts tested. Lower *p* values compare 2.5D fibrillar or 3D fibrillar FN matrices with 2D globular FN substrates. Upper *p* values compare 2.5D fibrillar with 3D fibrillar FN matrices. e–g), Adhesion forces of pKO‐*α*V/*β*1 (e), pKO‐*β*1 (f), or pKO‐*α*V (g) fibroblasts to PFA‐crosslinked 2D (2DX) globular FN substrates, PFA‐crosslinked 2.5D (2.5DX) fibrillar FN matrices, and PFA‐crosslinked 3D (3DX) fibrillar FN matrices at given contact times. Data representation as in (a–d). For reference adhesion forces of fibroblasts to respective non‐crosslinked FN substrates or matrices are given in gray. Bottom *p* values compare given adhesion forces with reference data. Middle *p* values compare 2.5DX fibrillar or 3DX fibrillar FN matrices with 2DX globular FN substrates. Upper *p* values compare 2.5DX fibrillar with 3DX fibrillar FN matrices. *P* values were calculated using two‐tailed Mann–Whitney test. AS indicates the slope of a linear regression and s.e. of the adhesion strengthening rate (pN s^−1^). *n* indicates the number of individual fibroblasts tested.

pKO fibroblasts established and strengthened their adhesion force to FN matrices and substrates minimally (**Figure** **2**a). In contrast, all fibroblast lines expressing FN‐binding integrins considerably strengthened adhesion to 2.5D and 3D fibrillar FN matrices and 2D globular FN substrates (Figure 2b–d). pKO‐*α*V/*β*1 fibroblasts established similar adhesion forces to the 2D globular FN substrate as pKO‐*α*V fibroblasts, and lower adhesion force than pKO‐*β*1 fibroblasts at contact times ≥50 s (Figure S2a, Supporting Information). This finding agrees with previous reports showing that *α*V*β*3 integrin outcompetes *α*5*β*1 integrin for FN‐binding in early cell adhesion.^[^ ^6^, ^14^ ^]^

Compared to 2D globular FN substrates, pKO‐*α*V/*β*1 fibroblasts established higher adhesion forces to 2.5D and 3D fibrillar FN matrices at contact times ≥120 s and ≥50 s, respectively. Also pKO‐*β*1 fibroblasts markedly increased adhesion forces to 2.5D and 3D fibrillar FN matrices at contact times ≥240 s and ≥120 s, respectively, compared to 2D globular FN substrates (Figure 2c; Figure S2b,c, Supporting Information). Importantly, pKO‐*α*V/*β*1 and pKO‐*β*1 fibroblasts established similar adhesion forces to 3D and 2.5D fibrillar FN matrices. This indicates that the printing process and the underlying glass did not affect the fibroblast adhesion initiation and strengthening to fibrillar FN matrices (Figure 2b,c; Figure S2b,c, Supporting Information). Thus, within the given time frame of our experiments, fibroblasts are mostly sensitive to the mechanical properties of the ≈23 µm thick 2.5D fibrillar FN matrices and not to the underlying support. This result is in line with a previous study in which mesenchymal stem cells detect the underlying glass substrate only when the thickness of the polyacrylamide gel coating is <20 µm.^[^ ^30^ ^]^ Further, pKO‐*α*V/*β*1 fibroblasts established lower adhesion force to fibrillar FN matrices than pKO‐*β*1 fibroblasts at contact times of 240 s and a higher adhesion force than pKO‐*α*V fibroblasts at contact times ≥120 s (Figure 2b–d; Figure S2a,d, Supporting Information). This finding indicates that *α*V*β*3 integrin also outcompetes *α*5*β*1 integrin to bind fibrillar FN faster and that *α*5*β*1 integrin contributes earlier to adhesion strengthening when both FN‐binding integrins are present.

Taken together, our results show that from the onset of contacting FN matrices *α*5*β*1 integrin‐expressing fibroblasts sense the FN fibrillarity and accelerate adhesion strengthening. Fibroblasts initiate and strengthen adhesion similarly to printed 2.5D and 3D fibrillar FN matrices, regardless of the vastly different mechanical properties of the underlying inorganic material.

### 2.3 Fibroblasts Further Accelerate Adhesion Initiation and Strengthening to Stiffened Fibrillar FN Matrices via *α*5*β*1 Integrin

Next, we asked whether the stiffness of FN matrices affects how fibroblasts initiate and strengthen adhesion. We crosslinked FN substrates and matrices with paraformaldehyde (PFA, 4% v/v), which does not alter the integrin‐ and heparin‐binding domains of FN.^[^ ^31^ ^]^ PFA crosslinking of 3D and 2.5D fibrillar FN matrices left their micro‐ and nano‐fibrillar architecture unaffected and stiffened both matrices by a factor of ≈5 to similar values (Figure S3, Supporting Information). SCFS showed that the crosslinking of 2D globular FN substrates did not affect the adhesion force and strengthening of all fibroblast lines (Figure 2e–g; Figure S4, Supporting Information). However, pKO‐*α*V/*β*1 fibroblasts considerably increased their adhesion force to crosslinked 3D and 2.5D fibrillar FN matrices at contact times ≥120 s and ≥20 s, respectively. Similarly, pKO‐*β*1 fibroblasts considerably increased adhesion force for all contact times and at contact times ≥50 s to crosslinked 3D and 2.5D fibrillar FN matrices, respectively. In contrast, the crosslinking of 3D and 2.5D fibrillar FN matrices did not affect the adhesion force of pKO‐*α*V fibroblasts. We verified that the increased cell adhesion force was mediated by *α*5*β*1 integrin by testing the adhesion of pKO‐*β*1 fibroblasts to fibrillar FN matrices crosslinked by methanol (Figure S5, Supporting Information), which also keeps the integrin‐ and heparin‐binding domains of FN intact.^[^ ^31^ ^]^

Together, our data shows that the stiffening of fibrillar FN matrices, irrespective whether they are freely spanning or printed on glass, induces fibroblasts to further accelerate adhesion strengthening. In fibroblasts, *α*5*β*1 integrin trigger this mechanosensitive adhesion regulation within seconds.

### 2.4 Fibrillar FN Triggers *α*5*β*1 Integrin and Syndecan‐4 Crosstalk Within Seconds

**Figure 3:**
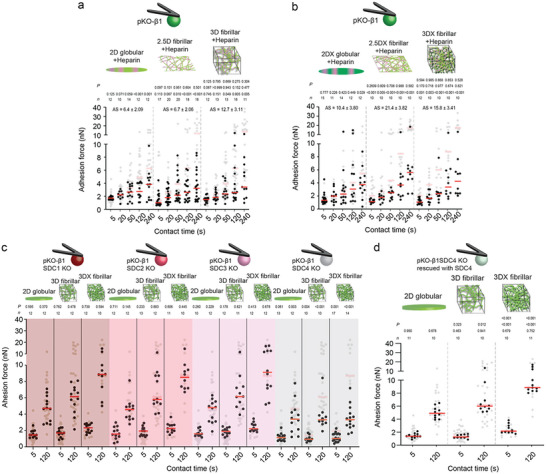
Syndecan‐4 crosstalks with *α*5*β*1 integrin to sense the fibrillarity and mechanical stiffness of FN. a,b) Adhesion forces of pKO‐*β*1 fibroblasts to heparin‐treated 2D globular FN substrates, 2.5D fibrillar FN matrices, and 3D fibrillar FN matrices a) without and b) with crosslinking. Reference adhesion forces of pKO‐*β*1 fibroblasts to non‐treated FN substrates or matrices are given in gray. Bottom *p* values compare given and reference data. Middle *p* values compare 2.5D fibrillar or 3D fibrillar FN matrices with 2D globular FN substrates. Top *p* values compare 2.5D fibrillar and 3D fibrillar FN matrices. AS indicates the slope of a linear regression and s.e. of adhesion strengthening rate (pN s^−1^). c) Adhesion forces of pKO‐*β*1 fibroblasts depleted from syndecan‐1 (SDC1 KO), syndecan‐2 (SDC2 KO), syndecan‐3 (SDC3 KO), or syndecan‐4 (SDC4 KO) to 2D globular, 3D fibrillar, and crosslinked 3D fibrillar (3DX) FN substrates. Adhesion forces of pKO‐*β*1 fibroblasts to respective FN substrates are given in gray as reference. *p* values compare given and reference data. d) Adhesion forces of pKO‐*β*1 SDC4 KO fibroblasts rescued with syndecan‐4 to 2D globular, 3D fibrillar, and crosslinked 3D fibrillar (3DX) FN substrates. Adhesion forces of pKO‐*β*1 fibroblasts to respective FN substrates are given in gray as reference. Bottom *p* values compare given and reference data. Middle *p* values compare 2.5D fibrillar or 3D fibrillar FN matrices with 2D globular FN substrates. Top *p* values compare 2.5D fibrillar and 3D fibrillar FN matrices. Dots represent adhesion forces of individual fibroblasts and red bars the median. *n* indicates the number of fibroblasts tested. *p* values were calculated using two‐tailed Mann–Whitney test.

To test whether syndecans participate in sensing the FN fibrillarity, we blocked heparin sulfate binding to FN by pre‐incubating 2D globular FN substrates, 2.5D fibrillar and 3D fibrillar FN matrices with heparin.^[^ ^6^ ^]^ Heparin did not alter the adhesion forces of pKO‐*α*V fibroblasts to the FN substrates and matrices tested (Figure S6a,b, Supporting Information). On the contrary, it slightly reduced the adhesion force of pKO‐*α*V/*β*1 and pKO‐*β*1 fibroblasts to 2D globular FN substrates at contact times of 240 s and ≥120 s, respectively (**Figure** **3**a; Figure S6c–e, Supporting Information). However, incubation of 3D and 2.5D fibrillar FN matrices with heparin considerably reduced the adhesion force of pKO‐*α*V/*β*1 and pKO‐*β*1 fibroblasts at contact times of ≥120 s and ≥50 s, respectively. Importantly, pKO‐*α*V/*β*1 and pKO‐*β*1 fibroblasts established similar adhesion forces to heparin‐incubated 2D globular FN substrates, 2.5D fibrillar and 3D fibrillar FN matrices for all contact times tested. The results show that the engagement of pKO‐*α*V/*β*1 and pKO‐*β*1 fibroblasts to heparin‐binding sites in fibrillar FN matrices increases *α*5*β*1 integrin‐mediated adhesion strengthening, thus indicating a potential involvement of syndecans in the regulation of adhesion initiation.

We next asked whether the heparin binding domains in FN also contribute to the sensing of the stiffness of fibrillar FN matrices. Thereto, we quantified the adhesion force of the fibroblast lines to heparin‐incubated and crosslinked 2D globular FN substrates, 2.5D fibrillar and 3D fibrillar FN matrices. Whereas heparin‐incubation of crosslinked 2D globular FN substrates and both fibrillar FN matrices did not affect the adhesion force and strengthening of pKO‐*α*V fibroblasts (Figure S7a,b, Supporting Information), heparin‐incubation of crosslinked 3D fibrillar and 2.5D fibrillar FN matrices considerably reduced the adhesion forces and strengthening of pKO‐*α*V/*β*1 and pKO‐*β*1 fibroblasts for all contact times (Figure 3b; Figure S7c–e, Supporting Information). The results show that pKO‐*α*V/*β*1 and pKO‐*β*1 fibroblasts must engage to heparin‐binding sites in FN to sense the stiffness of fibrillar FN matrices and to accelerate *α*5*β*1 integrin‐mediated adhesion strengthening.

To test whether any of the four syndecan family members crosstalks with *α*5*β*1 integrin to sense the fibrillarity and stiffness of FN, we depleted syndecan‐1 (pKO‐*β*1 SDC1 KO), syndecan‐2 (pKO‐*β*1 SDC2 KO), syndecan‐3 (pKO‐*β*1 SDC3 KO), or syndecan‐4 (pKO‐*β*1 SDC4 KO) from pKO‐*β*1 fibroblasts by CRISPR/Cas gene editing. We verified the unaltered *β*1 integrin surface expression level and similar cell size of all syndecan‐depleted fibroblast lines (Figure S8, Supporting Information). Next, we quantified the adhesion forces of each syndecan‐depleted pKO‐*β*1 fibroblast line to 2D globular FN substrates and to non‐crosslinked or crosslinked 3D fibrillar FN matrices at 5 and 120 s contact time (Figure 3c). The depletion of syndecan‐1, syndecan‐2, or syndecan‐3 did not change the adhesion forces of pKO‐*β*1 fibroblasts to any of the FN substrates and matrices tested. However, syndecan‐4 depletion reduced the adhesion force of pKO‐*β*1 fibroblasts slightly to 2D globular FN substrates at 120 s contact time and considerably to non‐crosslinked and crosslinked 3D fibrillar FN matrices at all contact times. Additionally, pKO‐*β*1 SDC4 KO fibroblasts established similar adhesion forces to 2D globular FN substrates and non‐crosslinked or crosslinked fibrillar FN matrices. To test whether the reduced adhesion forces of pKO‐*β*1 SDC4 KO fibroblasts to fibrillar FN was syndecan‐4 specific, we re‐expressed syndecan‐4 in pKO‐*β*1 SDC4 KO fibroblasts. The fibroblasts showed unaltered surface expression levels of *β*1 integrins, rescued surface expression levels of syndecan‐4, and cell sizes similar to pKO‐*β*1 SDC4 KO fibroblasts (Figure S8, Supporting Information). The re‐expression of syndecan‐4 restored the adhesion forces of pKO‐*β*1 SDC4 KO fibroblasts to levels observed for pKO‐*β*1 fibroblasts (Figure 3d). Importantly, heparin‐incubation of non‐crosslinked and crosslinked 3D fibrillar FN matrices did not affect the adhesion force of pKO‐*β*1 SDC4 KO fibroblasts, showing that depleting the syndecan‐4 binding to FN is the main effector for the reduction of adhesion forces to heparin‐incubated fibrillar FN (Figure S9, Supporting Information). Since pKO‐*β*1 SDC4 KO and pKO‐*β*1 fibroblasts adhering to heparin‐treated fibrillar FN matrices did not respond to the fibrillarity or stiffness of FN, we conclude that the potential ligand density differences in globular FN substrates and fibrillar FN matrices are not driving the adhesion strengthening of pKO‐*β*1 fibroblasts to fibrillar FN matrices.

Together, these results demonstrate that within the first 5 s of initiating adhesion, fibroblasts sense the stiffness of fibrillar FN matrices by a crosstalk between *α*5*β*1 integrin and syndecan‐4. The rapid crosstalk accelerates the adhesion strengthening of fibroblasts to fibrillar FN matrices and complements the mechanosensitive adhesion regulation of *α*5*β*1 on 2D FN‐fragment substrates.^[^ ^7a^ ^]^

### 2.5 Fibrillar FN Triggers Multiple Signaling Pathways to Accelerate Adhesion Initiation

**Figure 4:**
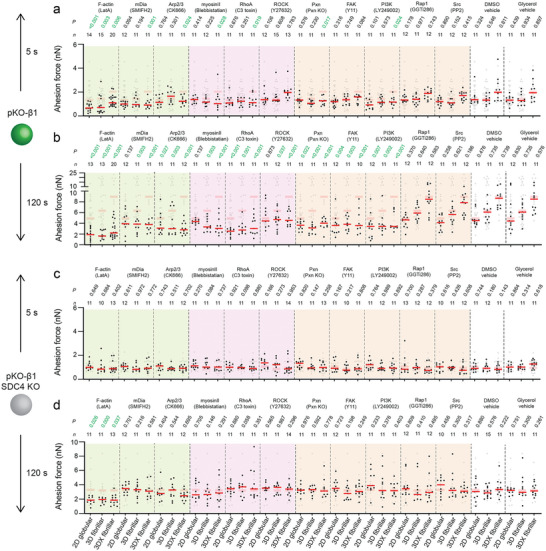
*α*5*β*1 integrin and syndecan‐4 crosstalk requires F‐actin polymerization, myosin II‐mediated contractility, and FAK signaling to strengthen adhesion in response to FN fibrillarity and stiffness. a–d) Adhesion forces of pKO‐*β*1 (a,b) or pKO‐*β*1 (c,d) SDC4 KO fibroblasts to 2D globular FN substrates, 3D fibrillar FN matrices, and crosslinked 3D (3DX) fibrillar FN matrices at 5 s (a,c) and 120 s (b,d) contact times in the presence of perturbations for F‐actin polymerization (1 µm LatA), mDia (20 µm SMIFH2), Arp2/3 (200 µm CK666), myosin II (20 µm blebbistatin), RhoA (2 µm C3 toxin), ROCK (10 µm Y27632), FAK (10 µm Y11), PI3K (10 µm LY249002), Rap1 (10 µm GGTi286), or Src (20 µm PP2). The effect of paxillin was tested using paxillin knock‐out (Pxn KO) fibroblasts. Dimethyl sulfoxide (DMSO) or glycerol (50% v/v) were tested as vehicle controls. Dots represent adhesion forces of individual fibroblasts and red bars the median. *n* indicates the number of fibroblasts tested. Adhesion forces of non‐crosslinked pKO‐*β*1 (a,b) or pKO‐*β*1 SDC4 KO (c,d) fibroblasts in the respective condition are given in gray for reference. *p* values compare given with reference data.

To understand which molecular pathways within fibroblasts connect the rapid sensing of the FN fibrillarity and stiffness with the accelerated adhesion strengthening, we aim to decipher the key players involved in the syndecan‐4–*α*5*β*1 integrin crosstalk. To this end, we chemically depolymerized F‐actin (latrunculin A, LatA), inhibited the actin polymerization machineries mDia (SMIFH2) or Arp2/3 (CK666), and actin contractility by perturbing myosin II (blebbistatin), RhoA (C3 toxin), or ROCK (Y27632). We also perturbed integrin related signaling proteins, including the focal adhesion kinase (FAK; Y11), phosphoinositide 3‐kinase (PI3K; LY294002), Rap1 (GGTi286), and Src (PP2). Using SCFS we characterized in the presence of the chemical perturbations whether pKO‐*β*1 and pKO‐*β*1 SDC4 KO fibroblasts can sense and respond to 2D globular FN substrates and to non‐crosslinked and crosslinked 3D fibrillar FN matrices at 5 or 120 s contact time (**Figure** **4**). Additionally, we quantified the adhesion force of paxillin‐depleted pKO‐*β*1 and pKO‐*β*1 SDC4 KO fibroblasts that had unaltered surface expression levels of *β*1 integrin and similar cell sizes (Figures S8d and S10, Supporting Information). At 5 s contact time, LatA‐treatment reduced the adhesion forces of pKO‐*β*1 fibroblasts to 2D globular FN substrate and to non‐crosslinked and crosslinked 3D fibrillar FN matrices. Additionally, the perturbation of mDia, Arp2/3, myosinII, RhoA, paxillin and PI3K reduced the adhesion forces of pKO‐*β*1 fibroblasts at 5 s contact time only to crosslinked 3D fibrillar FN matrices. At 120 s contact time, the perturbation of F‐actin, Arp2/3, RhoA and paxillin, FAK and PI3K reduced the adhesion forces of pKO‐*β*1 fibroblasts to 2D globular FN substrates and to non‐crosslinked or crosslinked 3D fibrillar FN matrices. However, we observed rather drastic differences in the magnitude at which the inhibitors reduced the adhesion forces of fibroblasts to 2D globular FN substrates or crosslinked and non‐crosslinked 3D fibrillar FN matrices. Additionally, the inhibition of mDia, myosinII, and ROCK reduced adhesion forces of pKO‐*β*1 fibroblasts to non‐crosslinked and crosslinked 3D fibrillar FN. Importantly, pKO‐*β*1 fibroblasts established similar adhesion forces to 2D globular and non‐crosslinked or crosslinked 3D fibrillar FN matrices upon the perturbation of the above mentioned signaling and adaptor proteins. Finally, inhibiting Src or Rap1 did not affect the adhesion force of pKO‐*β*1 fibroblasts to the globular FN substrate and fibrillar FN matrices. In pKO‐*β*1 SDC4 KO fibroblasts, only LatA‐treatment reduced the adhesion force to FN substrates and matrices at 120 s contact time, while none of the other perturbations altered the cell adhesion force irrespective of the contact time. Vehicles (DMSO or glycerol) did not affect the adhesion force of pKO‐*β*1 SDC4 KO or pKO‐*β*1 fibroblasts to any FN substrate and matrix (Figure 4).

In summary, upon the perturbation of the F‐actin network, mDia, Arp2/3, RhoA, ROCK, myosin II, FAK, paxillin, and PI3K, pKO‐*β*1 fibroblasts cannot differentiate between globular FN substrates and fibrillar FN matrices and cannot sense the stiffness of the FN matrices. Therefore, we conclude that these actin regulatory, adaptor and signaling proteins are involved in the crosstalk between *α*5*β*1 integrin and syndecan‐4, which enables fibroblast to sense and respond to the FN fibrillarity and stiffness. Interestingly, PI3K and FAK are linked to integrin activation during adhesion initiation by promoting the recruitment of kindlin and talin to integrins.^[^ ^7^, ^14^, ^32^ ^]^ This finding, which indicates that fibrillar FN accelerates the activation, recruitment and clustering of *α*5*β*1 integrin, is supported by a report showing that syndecan‐4 senses mechanical load and activates *α*5*β*1 integrin via RhoA/PI3K/kindlin cascade to strengthen focal adhesions at much longer time (>1 h) scales.^[^ ^10^ ^]^ While fibroblasts respond to fibrillar FN within 120 s, they respond to stiffened FN fibrils within 5 s. To sense the stiffness of FN fibrils, fibroblasts require the actin polymerizing mDia machinery and myosin II‐mediated contractility. Since mDia and myosin II are involved in the crosstalk, this indicates that syndecan‐4 signaling triggers actomyosin remodeling during early adhesion strengthening.

### 2.6 Fibroblasts Accelerate Migration on Fibrillar FN Matrices via *α*5*β*1 Integrin and Syndecan‐4

**Figure 5:**
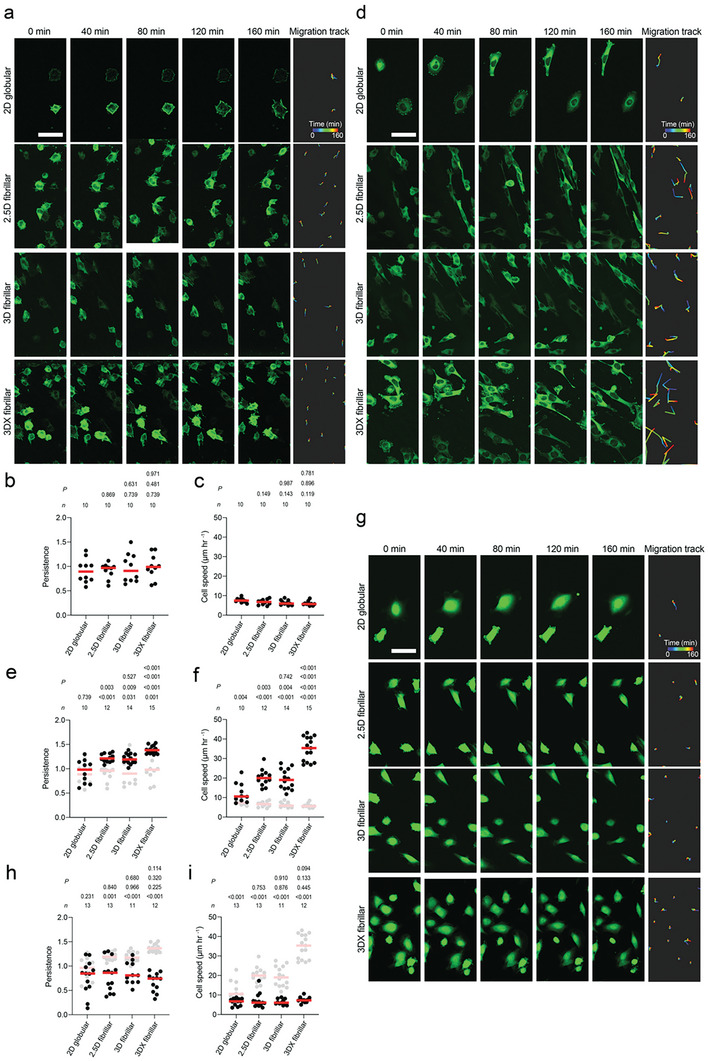
Fibroblasts regulate migration in response to FN fibrillarity via *α*5*β*1 integrin and syndecan‐4. a,d,g) Timelapse images of pKO‐*α*V fibroblasts expressing paxillin‐GFP (a), pKO‐*β*1 fibroblasts expressing paxillin‐GFP (d) and pKO‐*β*1 SDC4 KO (g) fibroblasts labeled with a live cell membrane staining CellTracker dye seeded on given FN substrates and fibrillar matrices. Scale bar, 50 µm. b,e,h) Persistence of fibroblasts as calculated by the slope of log–log plots of the mean square displacement versus the log time (Figure S11, Supporting Information). c,f,i) Migration speed of fibroblasts calculated from fluorescence images. Dots represent persistence and migration speed of fibroblasts on 2D FN substrates and different fibrillar FN matrices. Red bars indicate the median. In (b,c), top row *p* values compare 3D and 3DX fibrillar FN matrices and bottom, middle row *p* values compare 3D or 3DX fibrillar FN matrices with 2.5D fibrillar FN matrices, and bottom row *p* values compare 2D globular FN substrate to fibrillar FN matrices. In (e,f,) top *p* values compare 3D and 3DX fibrillar FN matrices, second row *p* values compare 3D or 3DX fibrillar FN matrices with 2.5D fibrillar FN matrices, third row *p* values compare 2D globular FN substrate to fibrillar FN matrices and bottom row *p* values compare persistence and cell speed of pKO‐*β*1 fibroblasts with those of pKO‐*α*V fibroblasts. h,i) Top row *p* values compare 3D and 3DX fibrillar FN matrices, second row *p* values compare 3D or 3DX fibrillar FN matrices with 2.5D fibrillar FN matrices, third row *p* values compare 2D globular FN substrate to fibrillar FN matrices and bottom row *p* values compare persistence and cell speed of pKO‐*β*1 SDC4 KO fibroblasts with pKO‐*β*1 fibroblasts. *n* indicates the number of FN substrates tested. *p* values were calculated using two‐tailed Mann–Whitney *t*‐tests.

Our adhesion initiation data shows that fibroblasts employ *α*5*β*1 integrin and syndecan‐4 to strengthen adhesion initiation to fibrillar FN matrices. Hence, we asked whether fibroblasts also employ *α*5*β*1 integrin and syndecan‐4 to migrate faster on fibrillar FN matrices. To evaluate the influence of fibrillar FN on cell migration, we seeded paxillin‐green fluorescent protein (GFP) expressing pKO‐*α*V, paxillin‐GFP expressing pKO‐*β*1, and pKO‐*β*1 SDC4 KO fibroblasts labeled with a live cell membrane dye on 2D globular FN substrates as well as 2.5D fibrillar FN, 3D fibrillar and crosslinked 3D fibrillar FN matrices and monitored their migration by time‐lapse confocal microscopy. Throughout the migration experiment, pKO‐*α*V fibroblasts exhibited flat morphologies on FN substrates and matrices (**Figure** **5**a). There was no significant difference in the persistency and migration speed of pKO‐*α*V fibroblasts across the FN substrates and matrices regardless of FN fibrillarity or stiffness (Figure 5b,c; Figure S11a, Supporting Information). While pKO‐*β*1 fibroblasts showed flat morphologies on 2D globular FN substrates, they elongated on 2.5D and 3D fibrillar FN matrices (Figure 5d) similar to fibroblasts observed in native 3D ECMs.^[^ ^1a^ ^]^ Compared to 2D globular FN substrates, pKO‐*β*1 fibroblasts on 2.5D and 3D fibrillar FN matrices also showed higher persistency and migration speed (Figure 5e,f; Figure S11b, Supporting Information). Upon stiffening the 3D fibrillar FN matrices, pKO‐*β*1 fibroblasts further increased persistency and migration speed. Compared to pKO‐*β*1 fibroblasts, pKO‐*β*1 SDC4 KO fibroblasts showed flat morphologies on 2D globular FN substrates and less elongated on non‐crosslinked and crosslinked fibrillar FN matrices (Figure 5g). The pKO‐*β*1 SDC4 KO fibroblasts also considerably reduced persistency and migration speed compared to pKO‐*β*1 fibroblasts (Figure 5h,i; Figure S11c, Supporting Information).

The results show that fibroblasts sense the fibrillarity and stiffness of FN via *α*5*β*1 integrin and syndecan‐4 to regulate migration on fibrillar FN matrices within the first few hours after attachment. This mechanosensitive migration behavior agrees with previous reports indicating that cell migration regulation depends on the substrate stiffness.^[^ ^33^ ^]^ However, such experiments are commonly conducted on supports having different stiffnesses and coated with 2D globular FN. We show that fibrillar FN matrices trigger a crosstalk between *α*5*β*1 integrin and syndecan‐4 within seconds of adhesion initiation, which accelerates long‐term cell migration. Interestingly, even after 160 min fibroblasts expressing exclusively *α*V‐class integrins do not adapt their migration behavior to fibrillar FN matrices, no matter whether the FN fibrils were stiffened or not.

### 2.7 Fibroblasts Maintain Proliferative Potential on Fibrillar FN Matrices via *α*5*β*1 Integrin and Syndecan‐4

**Figure 6:**
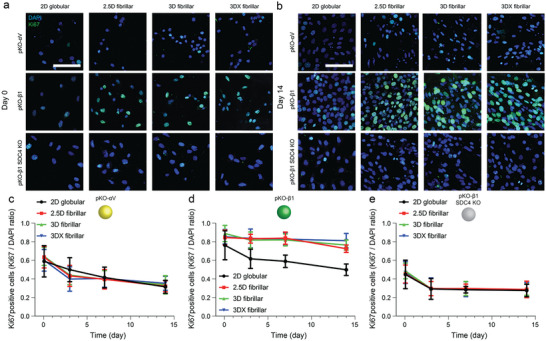
On fibrillar FN matrices fibroblasts maintain high proliferation via *α*5*β*1 integrin and sydecan‐4. a,b) Representative fluorescence images of fibroblasts on day 0 and 14. Blue and green represent DAPI and Ki67 staining, respectively. Scale bar, 50 µm. c–e) Ki67 positive (proliferative) fibroblasts as calculated by the ratio of Ki67 positive fibroblasts divided by DAPI stained pKO‐*α*V (c), pKO‐*β*1 (d), pKO‐*β*1 SDC4 KO (e) fibroblasts. *n* = 10 (two different regions of interest from five different samples per each condition). Symbols and error bars indicate the mean and standard deviation, respectively. Statistical analysis of proliferation data is shown in Figure S12, Supporting Information.

To test whether fibrillar FN matrices influence cell proliferation, we cultured pKO‐*α*V, pKO‐*β*1, and pKO‐*β*1 SDC4 KO fibroblasts 2D globular FN substrates as well as 2.5D fibrillar, 3D fibrillar, and crosslinked 3D fibrillar FN matrices for 1, 3, 7, and 14 days. The ratio of proliferative fibroblasts was assessed by Ki67 and DAPI staining (**Figure** **6**a,b). The ratio of Ki67 positive pKO‐*α*V fibroblasts decreased over 14 days on 2D globular FN substrates and 2.5D, non‐crosslinked 3D and crosslinked 3D fibrillar FN matrices independent of their stiffness (Figure 6c; Figure S12, Supporting Information). On the other hand, 2.5D, non‐crosslinked 3D, and crosslinked 3D fibrillar FN matrices maintained a high ratio of Ki67 positive pKO‐*β*1 fibroblasts from day 3 to day 14 compared to 2D globular FN substrates on which proliferation decreased with time. Stiffening of fibrillar FN did not influence the proliferation over 14 days (Figure 6d; Figure S12, Supporting Information). In all condition, the Ki67 positive cell ratio of pKO‐*β*1 fibroblasts was higher than that of pKO‐*α*V fibroblasts. Compared to pKO‐*β*1 fibroblasts, pKO‐*β*1 SDC4 KO fibroblasts showed significantly lower Ki67 positive cell ratio for all substrates over 14 days (Figure 6e; Figure S12, Supporting Information). Importantly, there was no difference in the Ki67 positive cell ratio of pKO‐*β*1 SDC4 KO fibroblasts among FN substrates and matrices.

Our results show that even after 14 days, the fibrillarity and stiffness of FN do not trigger fibroblasts, which exclusively engage FN with *α*V*β*3 integrins, to regulate their proliferation differently. On the other hand, the FN fibrils trigger the proliferation of fibroblasts that engage FN with *α*5*β*1 integrins and syndecan‐4. Apparently, this regulation is independent of the stiffness of the FN matrices. However, while the engineered fibrillar FN matrices remained largely intact after 14 days of fibroblast culture, we observed the beginning of their remodeling (Figure S13, Supporting Information). This observation suggests that after longer time intervals, fibroblast remodel the 3D fibrillar FN matrices even more and potentially also change their mechanical properties.^[^ ^34^ ^]^ Hence, we cannot exclude that the proliferation of fibroblasts also depends on the stiffness of the FN matrices, as shown before on ECM protein‐coated substrates.^[^ ^35^ ^]^ Similarly, to the accelerated adhesion strengthening and migration, the maintenance of highly proliferating fibroblasts expressing *α*5*β*1 integrins depends on the expression of syndecan‐4, indicating that fibrillar FN triggers multiple cellular functions over a long period.

## 3 Conclusion

**Figure 7:**
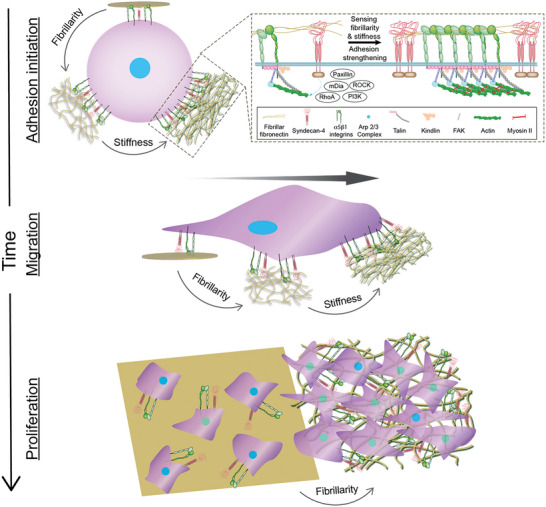
*α*5*β*1 integrin and syndecan‐4 sense the fibrillarity and mechanical stiffness of FN matrices and crosstalk to establish and regulate cell adhesion, migration, and proliferation. In fibroblasts *α*5*β*1 integrin and syndecan‐4 use multiple pathways to sense the fibrillarity and stiffness of FN. First, *α*5*β*1 integrin and syndecan‐4 bind to fibrillar FN and initiate cell adhesion. Within seconds, fibroblasts activate signaling pathways, which include mDia, Arp2/3, RhoA, paxillin, and PI3K, to strengthen adhesion to stiffer fibrillar FN by potentially polymerizing actin and myosin II‐mediated contraction. As adhesion matures, ROCK and FAK, in addition to the above‐mentioned signaling molecules, activate to further increase cell adhesion to both softer and stiffer fibrillar FN. Fibroblasts adhering to fibrillar FN matrices accelerate migration speed and enhance proliferation via *α*5*β*1 integrin and syndecan‐4. Although fibroblasts instantly sense fibrillar FN matrices via *α*5*β*1 integrin and syndecan‐4, both cell surface receptors are required to enhance migration speed ad proliferation for the time course of weeks. Upon stiffening of the fibrillar FN matrices the cell migration and proliferation further increase.

Here, we introduce a simple, versatile, and scalable platform to produce biomimetic fibrillar FN matrices. The 3D fibrillar matrices, which can be printed onto inorganic materials to increase their bio‐functionalization, accelerate fibroblast adhesion, migration, persistency, and maintain high proliferation potential. To differentiate between globular FN substrates and fibrillar FN matrices fibroblasts employ *α*5*β*1 integrin and syndecan‐4 within seconds to initiate adhesion. This rapid sensing of fibrillar FN matrices, which is further accelerated upon FN matrix stiffening, requires F‐actin polymerization and actomyosin contractility, paxillin, PI3K, and FAK (**Figure** **7**). With the initiation of cell adhesion, fibrillar FN matrices trigger an *α*5*β*1 integrin and syndecan‐4 crosstalk to enhance fibroblast migration and proliferation, which lasts for at least 14 days. Our results together with the recent literature^[^ ^21^, ^36^ ^]^ show that the genetic background as well as the expression levels of adhesion receptors and other membrane proteins determine whether and how fibroblasts respond to the fibrillarity of FN. Interestingly, syndecan‐4 is upregulated during wound healing to initiate *α*5*β*1 integrin‐mediated ECM fibril deposition and cell migration in the dermis.^[^ ^37^ ^]^ On the other hand, the loss of syndecan‐4 and/or *β*1 integrin leads to tissue stiffening in cardiac fibrosis.^[^ ^38^ ^]^

Hence, the rapid sensing of FN fibrillarity and stiffness mediated by *α*5*β*1 integrin–syndecan‐4 crosstalk during early adhesion and long‐term cell function may be related to how cells initiate and adapt adhesion to regulate their behavior in native tissues. Although we observe *α*5*β*1 integrin to participate in mechanosensing within 5 s, it should be noted that *α*V‐class integrins^[^ ^16a^ ^]^ and/or other syndecans^[^ ^10^ ^]^ may participate in the mechanosensing at later time points,^[^ ^13^, ^39^ ^]^ with different substrates (e.g., vitronectin)^[^ ^40^ ^]^ or with different cell types.^[^ ^37^, ^41^ ^]^

The understanding of how fibrillarity and stiffness of FN trigger specific cellular responses is an actively researched avenue. Studies report FN to accelerate^[^ ^31^, ^42^ ^]^ or decelerate^[^ ^43^ ^]^ cell migration and proliferation. This discrepancy possibly originates from the use of different FN states (globular FN, single FN fiber, or fibrillar FN matrices) and supports (glass, PDMS, synthetic polymer, or decellularized tissues with other undefined ECMs). The temporal and molecular dynamics of mechanosensing of FN by cells is not well known, necessitating further assessment. Hence, it is important to directly investigate how fibrillar FN can modulate cellular decisions over short and long time ranges differently compared to globular FN. Our investigation shows that fibroblasts continuously sense fibrillar FN matrices by both *α*5*β*1 integrin and syndecan‐4 to initiate and strengthen cell adhesion and to guide cell migration, persistency and proliferation. Lastly, our platform to engineer fibrillar FN matrices that rapidly accelerate integrin‐mediated cell adhesion, migration, and proliferation provides fundamental insights to better design regenerative biomaterials^[^ ^44^ ^]^ and in vitro tissue models.^[^ ^45^ ^]^

## 4 Experimental Section

### Engineering Globular and Fibrillar Fibronectin Substrates

3D fibrillar fibronectin (FN) matrices were fabricated using hydrodynamic shear force, as described.^[^ ^21a^ ^]^ First, microporous grids, square mesh holes with 400 µm thickness and 200 µm edge length, were outsourced and 3D printed using stereolithography with MicroFine materials (Proto Labs). The microporous grid was placed at the center of a 0.5 mL microcentrifuge tube and incubated with bovine full‐length FN solution (300 µL, 200 µg mL^−1^, Sigma Aldrich). The tube was rotated using a rotisserie rotator (Stuart) at 30 °C and 20 rpm for 2 h, which created hydrodynamic shear forces at the interface between air, microporous grid, and FN solution to induce FN fibrillogenesis. Hydrodynamically‐induced 3D fibrillar FN matrices were washed three times by phosphate‐buffered saline (PBS, Life Technologies) and stored in PBS at 4 °C before use. The parameters used to engineer fibrillar FN matrices were optimized in terms of grid geometry, protein concentration, rotation speed, time, and temperature compared to previous publications.^[^ ^21^ ^]^ The shear stress applied in the method was roughly estimated to range from 1 to 10 Pa.^[^ ^46^ ^]^ 2.5D fibrillar FN matrices were produced by contact‐printing 3D fibrillar FN matrices onto glass similar to traditional microcontact printing techniques.^[^ ^22^ ^]^ Specifically, the microporous grid with 3D fibrillar FN matrices was placed and contacted with a glass‐bottomed Petri dish (FD35, WPI) for 10 s. While the physical contact, the 3D fibrillar FN matrix was transferred to the dish making a 2.5D fibrillar FN matrix. As a control, 2D globular FN substrate was fabricated by incubating FN solution (50 µg mL^−1^) on a glass‐bottomed Petri dish at 4 °C overnight. For crosslinking, FN substrates or matrices were incubated with paraformaldehyde (PFA, 4% v/v) in PBS at 37 °C for 20 min or ice‐cold methanol (Sigma) for 5 min, followed by washing three times by PBS. Before experiments, glass surfaces were passivated with RGD‐deleted FN fragments (FNIII7‐10∆RGD, 50 µg mL^−1^),^[^ ^6^ ^]^ which was produced from plasmid pET15b‐FNIII7‐10 in *Escherichia coli* BL21 (DE3) pLysS as described,^[^ ^47^ ^]^ at 4 °C overnight. For detergent insolubility test, engineered 3D fibrillar FN matrices were treated with 1% deoxycholate (DOC) solution in PBS at 37 °C for a week. After DOC treatment, substrates were fluorescently stained by anti‐FN (full‐length FN, Abcam, ab2413) with 1:100 dilution in PBS at room temperature for 1 h. After incubation, substrates were washed twice by PBS and incubated with Alexa Fluor 488 goat anti‐rabbit IgG antibody at room temperature for 1 h. After incubation, substrates were washed twice by PBS and imaged using an inverted spinning disk confocal microscopy with 40× objective (W1‐SoRa Eclipse Ti2‐E, Nikon).

### FN Immunostaining and Confocal Microscopy

Engineered FN substrates were incubated with anti‐FN (full‐length FN, Abcam, ab2413) and FN IST‐9 (Abcam, ab6328) with 1:100 dilution in PBS at room temperature for 1 h. After incubation, substrates were washed twice by PBS and incubated with Alexa Fluor 488 goat anti‐rabbit IgG (for full‐length FN) and Alexa Fluor 594 goat anti‐mouse IgG (for FN IST‐9) antibodies at room temperature for 1 h. After incubation, substrates were washed twice by PBS and imaged using an inverted spinning disk confocal microscopy with 40× objective (W1‐SoRa Eclipse Ti2‐E, Nikon).

### Scanning Electron Microscopy (SEM)

Briefly, 2D globular, 2.5D fibrillar, and 3D fibrillar FN substrates were washed by DI water which was exchanged with ethanol. Samples were then dried using a critical point dryer (Safematic CDS Prototyp 5.5) and sputter‐coated with 20 nm thickness of Au (EM ACE600, Leica). Afterward, samples were imaged by SEM (ESEM XL30, Philips).

### Atomic Force Microscopy (AFM) Imaging

To characterize globular FN substrates, an AFM (NanoWizzard II, JPK Instruments) mounted on an inverted microscope (Axio Observer Z1, Zeiss) was used. AFM imaging was performed using contact mode with a V‐shaped cantilever (SNL, Brucker) having a nominal spring constant of 0.06 N m^−1^. First, an area of 20 × 20 µm^2^ was imaged at a line rate of 1 Hz using 512 × 512 pixels. The imaging force was kept <2 nN and compensated for the thermal drift. AFM scratching was on an area of 10 × 10 µm^2^ by applying a force of ≈60 nN at a line rate of 15 Hz. After performing ten scratches, the scanning direction of the AFM cantilever was rotated 90° and another ten scratches were performed. Thereafter, the original 20 × 20 µm^2^ surface area was reimaged using the AFM imaging settings described above.

### FN Stiffness Measurements

A silica bead (Kisker, Biotech, 5 µm diameter bead) was glued to the free end of tipless microcantilevers (NP‐O A, Bruker) using UV glue (Dymax) and cured under UV light for 20 min. Cantilevers with beads were plasma‐treated for 5 min using a plasma cleaner (Harrick Plasma) to ensure a clean surface and mounted on the cleaned fluid cell (probe holder) of the AFM (Bioscope resolve, Bruker). Force–distance curves were collected with the beaded cantilevers approaching onto the substrate until a setpoint of 1 nN was reached, with a constant approach and retract velocities of 10 µm s^−1^. Each approach force–distance curve was baseline and tilt corrected using the non‐contact region of the curve. The linear part of contact region in the approach curve was fitted with the Hertz model to estimate the Young's modulus of the sample^[^ ^48^ ^]^ using an analysis software (Nanoscope analysis v 1.80).

### Cell Line Engineering and Culture

pKO‐*α*V/*β*1, pKO‐*β*1, pKO‐*α*V (all ref. [15b]), pKO‐*β*1 SDC1 KO, pKO‐*β*1 SDC2 KO, pKO‐*β*1 SDC3 KO, and pKO‐*β*1 SDC4 KO fibroblasts were cultured on FN‐coated tissue culture flasks (Jet Biofil) with DMEM GlutaMAX (Gibco‐Life technologies) containing fetal bovine serum (FBS, 10% w/v, Sigma Aldrich), penicillin (100 U mL^−1^), and streptomycin (100 µg mL^−1^, both Thermo Fisher Scientific).

To deplete syndecans in pKO‐*β*1 fibroblasts, CRISPR/Cas9 was employed as described.^[^ ^49^ ^]^ Briefly, single‐guide RNAs (sgRNAs) for two different exons were designed using CHOPCHOP (https://chopchop.cbu.uib.no)^[^ ^50^ ^]^ for *Sdc1*, *Sdc2*, *Sdc3*, and *Sdc4* genes in *Mus musculus* genome (mm10/GRCm38). sgRNAs were selected based on higher rank, low self‐complementarity, low off‐target score, and efficiency score. The sgRNAs (with underlined PAM sequences; 5′‐NGG‐3′) that were designed targeted two separate exons on these genes. For *Sdc1*, exon 3 (chr12:8 790 825) [5′‐ACTGCCAATCAGCTTCCCGCAGG‐3′] and exon 4 (chr12:8 791 334) [5′‐ATTCCGCAGGCCGGGCTCTACGG‐3′] were targeted.

For *Sdc2*, we designed sgRNAs against exon 2 (chr15:33 017 104)

[5′‐AACAGAGCTGACATCCGATAAGG‐3′] and exon 5 (chr15:33 032 428)

[5′‐TGCTATTGGTGTACCGCATGCGG‐3′]. For *Sdc3*, exon 2 (chr4:130 816 855)

[5′‐GGCGCAATGAGAACTTCGAGAGG‐3′] and exon 5 (chr4:130 822 751)

[5′‐GTACGTGACGCTTGCCTGCTTGG‐3′] were targeted again. Exon 2 (chr2:164 431 230) [5′‐AGCATCTTCGTCGTCGGGGAGGG‐3′] and exon 5 (chr2:164 426 086) [5′‐TCATGCGTAGAACTCATTGGTGG‐3′] of *Sdc4* gene were targeted. Briefly, the forward and reverse DNA oligomers encoding sgRNAs were phosphorylated using T4 polynucleotide kinase enzyme (New England BioLabs) and then hybridized. Following a BpiI (Thermo Fisher Scientific) mediated digestion of backbones containing Cas9 and fluorescent reporter protein expression cassettes (pSpCas9‐2A‐BFP and pSpCas9‐2A‐GFP), sgRNA hybrids were ligated to the linearized backbones using T4 DNA ligase (New England BioLabs). pKO‐*β*1 fibroblasts were then transfected using lipofectamine 3000 (Invitrogen) following the product manual. After 48 h of transfection, green fluorescence protein (GFP) and blue fluorescence protein (BFP) double positive fibroblasts were sorted by a fluorescence activated cell sorter (FACS; SONY MA900) and cultured on FN‐coated culture dishes. Fibroblasts lacking the respective syndecan receptors were sorted using SONY MA900 sorter and propagated. Loss of respective syndecan expression was verified using immunolabeling and flowcytometric analysis (see Experimental Section).

In order to reintroduce the Sdc4 receptor in pKO‐*β*1 SDC4 KO fibroblasts, fibroblasts were cultured on FN coated 6 well plate. After attaining a confluency of ≈70%, the fibroblasts were transfected with a pCMV3‐mSDC4 plasmid (Sino Biological Inc., MG50726‐UT) using Lipofectamine 3000. This plasmid contained mouse Sdc4 cDNA (RefSeq NM_01 1521.2) inserted into a pCMV3‐untagged vector with a hygromycin resistance gene. The transfection medium was washed off after ≈20 h and replaced with culture medium containing hygromycin B (50 µg mL^−1^, Millipore, 400 053). The hygromycin selection was continued for 3 days with daily replenishment of new antibiotic containing culture medium. After 3 days of selection, the surviving fibroblasts were stained with phycoerythrin (PE)‐labeled SDC4 (1:50, Abcam, ab279590) antibody along with rabbit IgG control (1:50, Abcam, ab209478) antibody for flow cytometry (see **Methods “*Flow cytometry*”** section). Only cells with PE signal similar to the parental pKO‐*β*1 fibroblasts were sorted using Sony MA‐900, expanded, and further used for SCFS, cell size, and *β*1 integrin expression measurements.

To deplete paxillin in pKO‐*β*1 fibroblasts and pKO‐*β*1 Sdc4KO fibroblasts, a CRISPR/Cas9 strategy was employed as described above in case of syndecan knockout production. Single‐guide RNAs (sgRNAs) for two different exons were designed using CHOPCHOP (https://chopchop.cbu.uib.no) for paxillin in *Mus musculus* genome (mm10/GRCm38). The sgRNAs (with underlined PAM sequences; 5′‐NGG‐3′) that we designed targeted two separate exons on paxillin. For paxillin, exon 2 (chr5:115 544 490) [5′‐ CGTGCCATTGAGGGCCTCGCTGG‐3′] and exon 8 (chr5:115 552 099) [5′‐ GTAAGGTCGTGACCGCCATGGGG‐3′] were targeted.

After 48 h of transfection, GFP and BFP double positive cells were sorted using a FACS (SONY MA900) and cultured on FN‐coated culture dishes. Loss of paxillin was confirmed using western blotting using a monoclonal anti‐paxillin antibody (1:2500, abcam, ab32084) with glyceraldehyde 3‐phosphate dehydrogenase (GAPDH, 1:2500, Cell Signaling Technology, clone 14C10; 2118S) as loading control. Both primary antibodies were detected by using a horseradish peroxidase (HRP)‐conjugated goat anti rabbit secondary antibody (1:2500, BioRad, 170–6515). For immunostaining, fibroblasts (≈80% confluency) were fixed by PFA (4% v/v) with Triton X‐100 (0.05% v/v, Sigma) for 10 min at room temperature, washed twice by PBS, and blocked with BSA (5% w/v) for 1 h at room temperature. Fixed cells were incubated with the same monoclonal anti‐paxillin antibody used above (1:250 in PBS) for 1 h at room temperature followed by secondary antibody incubation (Alexa Fluor 488 goat anti‐rabbit IgG) and 4′,6‐diamidino‐2‐phenylindole (DAPI) at room temperature for 1 h. After incubation, substrates were washed twice by PBS and imaged using an inverted spinning disk confocal microscopy with 40× objective (W1‐SoRa Eclipse Ti2‐E, Nikon).

### Single‐Cell Force Spectroscopy (SCFS)

For SCFS, an AFM (CellHesion200, JPK Instruments) and a motorized stage (JPK Instruments) was used within a heat chamber (Life Imaging Services) to sustain ambient temperature at 37 °C during SCFS experiments. Tipless, V‐shaped, 200 µm long silicon nitride cantilevers (NP‐O, Bruker) with a nominal spring constant of 0.06 N m^−1^ was used. Prior to the experiments, the spring constant of each cantilever was determined using the thermal noise method.^[^ ^51^ ^]^ Cantilevers were cleaned using a plasma cleaner (PDC‐32G, Harrick Plasma) and incubated with concanavalin A (ConA, 2 mg mL^−1^, Sigma Aldrich) in PBS at 4 °C overnight, as described.^[^ ^52^ ^]^

Fibroblasts (≈80% confluency) were serum‐starved with DMEM GlutaMAX, penicillin (100 U mL^−1^), and streptomycin (100 µg mL^−1^) overnight, washed with PBS, and incubated with trypsin/EDTA (200 µL, 0.25% w/v, Sigma) for 2 min. Detached cells were resuspended in SCFS media (DMEM supplemented with HEPES (20 mm), penicillin (100 U mL^−1^), and streptomycin (100 µg mL^−1^) containing FBS (1% w/v), and pelleted. Cells were finally resuspended in serum‐free SCFS media. After trypsinization, fibroblasts were recovered for at least 30 min.^[^ ^47^ ^]^ FN substrates were submerged with SCFS media. Recovered fibroblasts were added to FN substrates. Rounded fibroblasts with similar size for SCFS experiments were optically monitored and selected. A calibrated ConA‐coated cantilever was approached to single fibroblasts at a speed of 10 µm s^−1^ until reaching a contact force of 5 nN. The cantilever was maintained at constant height for 5 s before retracting the cantilever and the bound fibroblast from the substrate by >50 µm. After retraction, the fibroblast was allowed to firmly attach to the cantilever for 5 min. Fibroblast adhesion forces were measured by approaching cantilever‐bound single fibroblasts to the substrate at an approach speed of 5 µm s^−1^ until reaching a contact force of 1 nN. Thereafter, the cantilever height was kept constant for the contact times of 5, 20, 50, 120, or 240 s. Then, the cantilever was retracted at a speed of 5 µm s^−1^ for a distance of >90 µm to fully detach the fibroblast from the substrate. The maximum downward deflection of the cantilever quantified the adhesion force of the cantilever‐bound fibroblasts. Cells were recovered from the detachment procedure for longer than the contact time before testing adhesion forces for a different contact time unless morphological changes such as spreading were discovered. At least ten fibroblasts per condition were tested to get statistically relevant results. Adhesion forces were determined from force–distance curves by using the JPK data analysis software, while adhesion force strengthening was calculated as the slope of a linear fit to the adhesion force over contact times using Prism software (GraphPad).

### Cell Migration and Proliferation Analysis

pKO‐*α*V and pKO‐*β*1 fibroblasts expressing lifeact‐mCherry and paxillin‐GFP (ref. [15b]) as well as pKO‐*β*1 Sdc4KO fibroblasts were cultured on FN‐coated tissue culture flasks with DMEM GlutaMAX containing FBS (10% w/v), penicillin (100 U mL^−1^), and streptomycin (100 µg mL^−1^). Upon reaching ≈80% confluency, the fibroblasts were washed with PBS and incubated with trypsin/EDTA (0.25% w/v, Sigma) for 2 min. pKO‐*β*1 Sdc4KO fibroblasts were incubated with a live cell membrane staining CellTracker dye (ThermoFisher) as per the manufacturer's manual. For migration assay, detached fibroblasts were seeded and allowed to attach to the FN substrate for 30 min in DMEM supplemented with FBS (1% w/v). Afterward, samples were mounted onto a point scanning confocal microscopy (LSM 980, Zeiss) with a 40 × objective (LD C‐Apochromat 40×/1.1 water immersion objective, Zeiss). Temperature and CO_2_ level were maintained at 37 °C and 5% during imaging by the environmental control system (Zeiss), respectively. Time‐lapse images were recorded every 20 min for 160 min. Imaris software (Oxford Instruments) was used to quantify mean square displacement and migration speed of the fibroblasts from the time‐lapse images. For proliferation assay, cells were cultured for 1, 3, 7, and 14 days and were then fixed by PFA (4% v/v) with Triton X‐100 (0.05% v/v, Sigma) for 10 min at room temperature, washed twice by PBS, and blocked with BSA (5% w/v) for 1 h at room temperature. Fixed cells were incubated with a monoclonal anti‐Ki67 antibody conjugated with FTIC (1:250 in PBS, Invitrogen, 11‐5698‐82) and DAPI for 1 h at room temperature at room temperature for 1 h. After incubation, substrates were washed twice by PBS and imaged using onto a point scanning confocal microscopy (LSM 980, Zeiss) with a 40× objective (LD C‐Apochromat 40×/1.1 water immersion objective, Zeiss).

### Perturbation

Target proteins were perturbed by incubating fibroblasts at 37 °C for 30 min in SCFS media with inhibitors as follows; latrunculin A (LatA, 1 µm, Sigma), SMIFH2 (20 µm, Merck Millipore), CK666 (200 µm, Tocris Bioscience), blebbistatin (20 µm, Sigma), Y27632 (10 µm, Sigma), Y11 (10 µm, Tocris Bioscience), LY249002 (10 µm, Cell Signaling Technology), GGTi286 (10 µm, Merck Millipore), or PP2 (20 µm, Tocris Bioscience) in DMSO. Fibroblasts were incubated with C3 toxin (2 µm, cytoskeleton) in glycerol (50% v/v, PanReac AppliChem) at 37 °C for 3 h in SCFS media. All inhibitors were present in the given concentrations during SCFS experiments. DMSO and glycerol (50% v/v) were tested as carrier controls to confirm no effect on adhesion forces from carrier solvents. For heparin treatment, FN substrates and matrices as well as fibroblasts were incubated with a saturating concentration^[^ ^6^ ^]^ of heparin (100 µg mL^−1^, Sigma) at 37 °C for 1 h in SCFS media before SCFS measurements. Heparin was present at the given concentrations during SCFS experiments.

### Flow Cytometry

Fibroblasts were cultured on FN‐coated 6 well plates up to ≈80% confluency. Cells were detached from 6 well plates using trypsin/EDTA (0.25% w/v) at 37 °C for 2 min. The detached fibroblasts were resuspended in SCFS media with FBS (1% w/v) and recovered from detachment process at 37 °C for at least 30 min. After recovery, cells were pelleted and 10^6^ fibroblasts were resuspended in flow cytometry buffer (100 µL, EDTA (2 mm) and BSA (2% w/v) in PBS) containing antibodies as follows; phycoerythrin (PE)‐labeled integrin subunit *β*1 (1:6, BioLegend, 102 208), SDC1 (1:10, R&D systems, FAB2966P) with rat IgG control (1:10, R&D systems, IC005P), or SDC4 (1:50, Abcam, ab279590) with rabbit IgG control (1:50, Abcam, ab209478) antibodies/Alexa Fluor 647‐conjugated SDC2 (1:20, R&D systems, FAB6585R) with sheep IgG control (1:20, R&D systems, IC016R) antibodies at 4 °C for 1 h. For SDC3, 10^6^ fibroblasts were incubated with SDC 3 (1:400, ThermoFisher, PA5‐47377) with Goat IgG control (1:400, ThermoFisher, 02–6202) antibodies in flow cytometry buffer at 4 °C for 1 h, washed by flow cytometry media twice, and incubated with Alexa Fluor 488 rabbit anti‐goat IgG (Invitrogen, A11078) at 4 °C for 1 h. After incubation, fibroblasts were washed by flow cytometry buffer twice, followed by measuring their fluorescence intensity employing LSRFortessa (BD AG). The flow cytometry data was analyzed by using FlowJo (v10, BD AG).

### Cell Size Analysis

Cells were detached and resuspended in SCFS media followed by 30 min recovery at 37 °C. After recovery, trypan blue (ThermoFisher) was added to the cell suspension solution at 1:1 ratio which was then loaded to a cell counting chip. The size of live cells was calculated using Countess II FL (Life Technologies).

### Statistical Analysis

All statistical analysis was characterized by using Prism (GraphPad Software). Unpaired, nonparametric two‐tailed Mann‐Whitney *t*‐tests were applied to evaluate *p* values and statistical significance. Linear regression analysis with a two‐tailed extra sum‐of‐squares *F*‐test was used to test adhesion strengthening of adhesion forces at different contact times. *P* values lower than 0.05 were considered as significant.

## Conflict of Interest

The authors declare no conflict of interest.

## Author Contributions

S.A., N.S., and D.J.M. conceived and designed the study. S.A. performed most of the experiments. U.S. engineered syndecan KO and paxillin KO fibroblast lines and performed flow cytometry and western blot experiments. S.A. and N.S. analyzed SCFS data. S.A. and U.S. analyzed flow cytometry data. K.C.K. performed stiffness measurements. N.S. performed AFM imaging with scratch assay. S.A., N.S., and D.J.M. evaluated experimental progress and data. All authors discussed the experiments and wrote the manuscript.

## Supporting information

Supporting Information

## Data Availability

The data that support the findings of this study are available from the corresponding author upon reasonable request.
