# Supplementary material for: Engineered Biomimetic Fibrillar Fibronectin Matrices Regulate Cell Adhesion Initiation, Migration, and Proliferation via α5β1 Integrin and Syndecan‐4 Crosstalk
Source: Adv Sci (Weinh). 2023 Jun 25;10(24):2300812. doi: 10.1002/advs.202300812 (PMC10460904; doi:10.1002/advs.202300812)
Supplement: Supplementary file 1 — Supporting Information [file ADVS-10-2300812-s001.pdf]

ADVANCED SCIENCE
Open Access

Supporting Information

for *Adv. Sci.*, DOI 10.1002/advs.202300812

Engineered Biomimetic Fibrillar Fibronectin Matrices Regulate Cell Adhesion Initiation, Migration, and Proliferation via $\alpha 5\beta 1$ Integrin and Syndecan-4 Crosstalk

Seungkuk Ahn, Upnishad Sharma, Krishna Chaitanya Kasuba, Nico Strohmeyer* and Daniel J. Müller**

Supporting Information

Engineered biomimetic fibrillar fibronectin matrices regulate cell adhesion initiation, migration, and proliferation *via* α5β1 integrin and syndecan-4 crosstalk

Seungkuk Ahn[1], Upnishad Sharma[1], Krishna Chaitanya Kasuba[1], Nico Strohmeyer[1*], and Daniel J. Müller[1*]*

Supplementary Figures

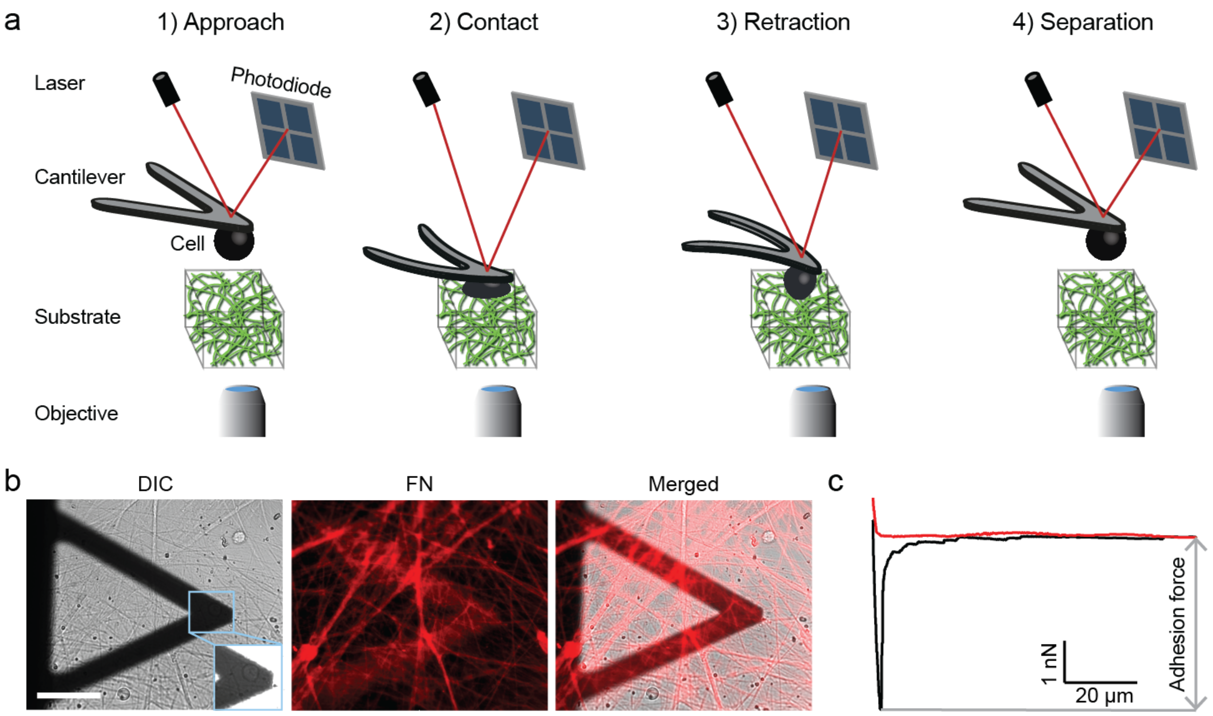

Figure S1. Atomic force microscopy (AFM)-based single-cell force spectroscopy (SCFS) setup to quantify the adhesion force of single fibroblasts. a) Schematic illustration of SCFS setup and approach. 1) A single fibroblast attached to a cantilever is approached to the substrate until cell and substrate get into contact. 2) Then, the cell is kept in contact with the substrate for a pre-set contact time. Thereby the cantilever height is kept constant. 3) After the contact time has passed, the cantilever is retracted to 4) fully separate fibroblast and substrate. b) Differential interference contrast (DIC) image of the AFM cantilever with a single fibroblast attached (inset), fluorescence image of a 3D fibrillar FN matrix (red, full-length FN antibody) and merged images. Scale bar, 100 µm. c) Representative force-distance curve of a SCFS experiment, which was recorded upon approaching (red curve) and retracting (black curve) a single fibroblast to and from the 3D fibrillar FN matrix, respectively. SCFS was conducted identically for 2D globular FN substrates and for 2.5D and 3D fibrillar FN matrices.

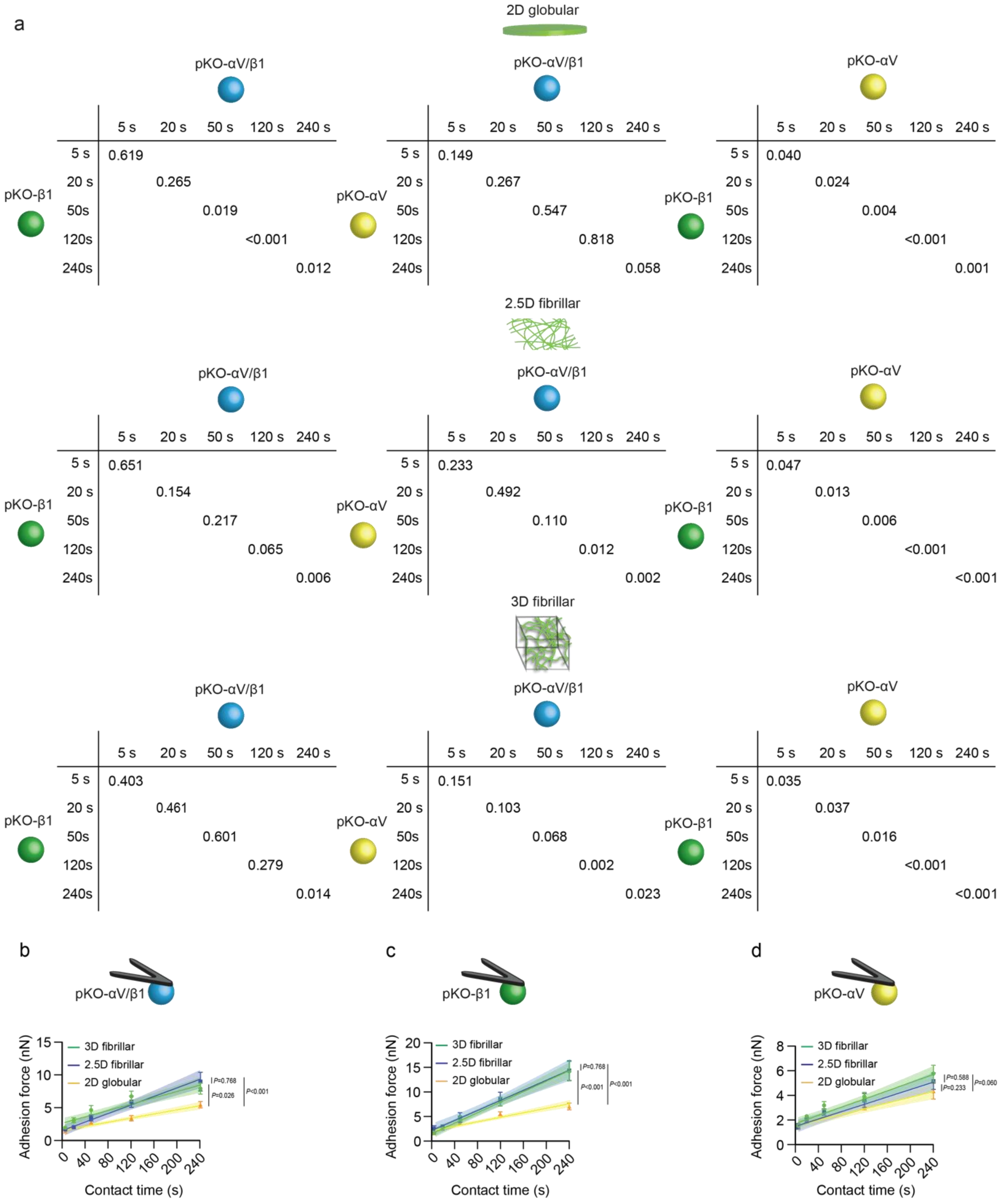

Figure S2. Statistical and adhesion strengthening analysis of fibroblast adhesion. a) Statistical analysis of each cell lines per each substrates/matrices. Data is taken from SCFS experiments in Figure 2. *P* values were calculated using the two-tailed Mann-Whitney test. b-d) Analysis of the adhesion strengthening of fibroblasts to 2D globular FN substrates and to 2.5D and 3D fibrillar FN matrices. Linear regression analysis of the adhesion force of (b) pKO-αV/β1, (c) pKO-β1, or (d) pKO-αV to 2D globular FN substrates, 2.5D fibrillar FN matrices, and 3D fibrillar FN matrices. Data is taken from SCFS experiments in Figure 2. Lines show linear fits of the data with shaded areas representing their 95% confidence intervals. The slope of each linear fit is used to determine the adhesion strengthening of fibroblasts. Dots represent mean adhesion force and error bars s.e.. *P* values were calculated using the two-tailed Mann-Whitney test.

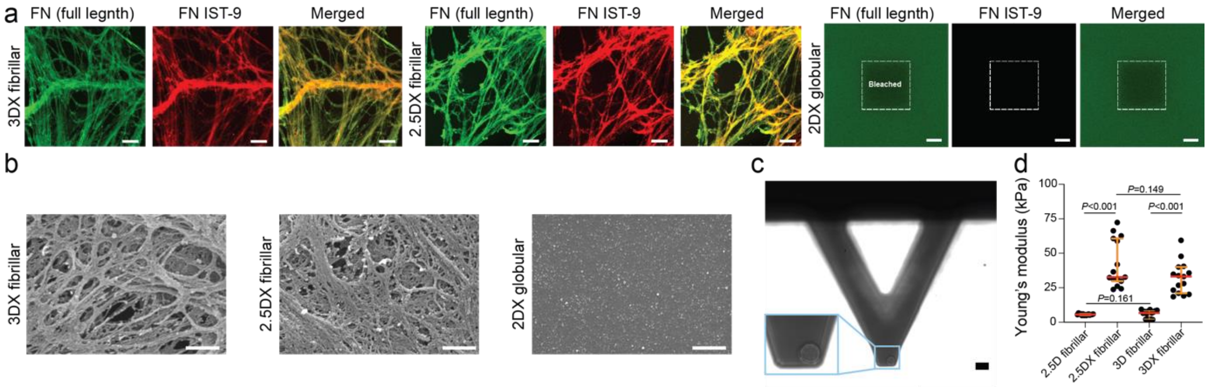

Figure S3. Characterization of crosslinked globular FN substrates and fibrillar FN matrices. a) Representative fluorescence images of crosslinked 2D (2DX) globular FN substrates, 2.5D (2.5DX) fibrillar FN matrices and 3D (3DX) fibrillar FN matrices. Full-length FN is shown in green (full-length FN antibody) and unfolded FN in red (FN IST-9). Dashed boxes in 2DX globular FN substrate indicate the photobleached area to distinguish fluorescent signal and background. Scale bars, 20 µm. b) Representative scanning electron microscopy (SEM) images of crosslinked FN substrates and matrices. Scale bars, 1 µm. c) Representative bright field image of a bead-bound cantilever (bead diameter ≈ 6.5 µm (mean)) used for stiffness measurements. Scale bar, 10 µm. d) Stiffness of 2.5D and 3D fibrillar FN matrices before and after chemical crosslinking. Dots represent stiffness of individual matrices. Red bars indicate the mean stiffness and orange bars the 95% confidential interval. *P* values were calculated using the two-tailed Mann-Whitney test.

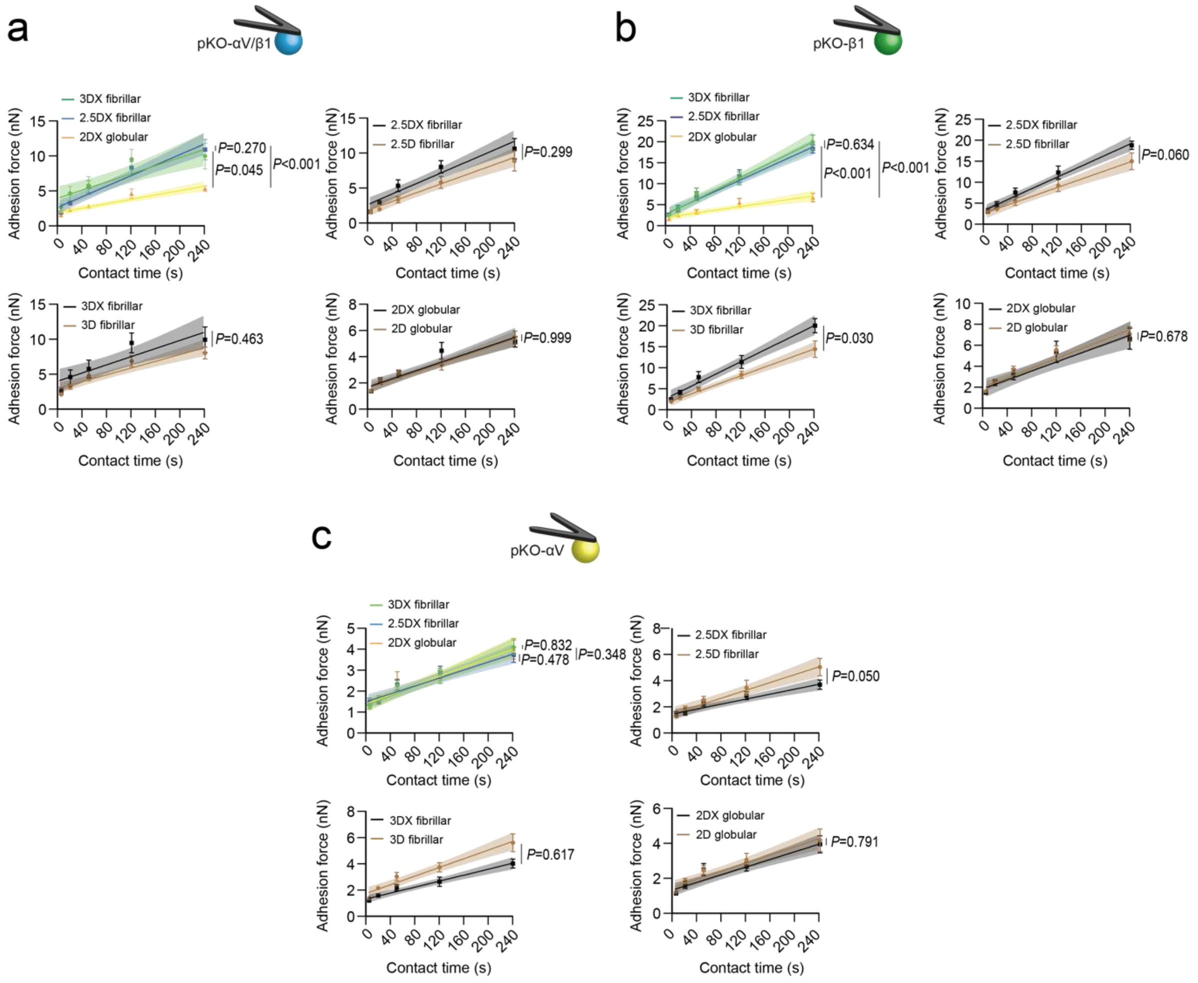

Figure S4. Adhesion strengthening of fibroblasts to non-crosslinked and to crosslinked 2D globular FN substrates and 2.5D and 3D fibrillar FN matrices. a-c) Linear regression analysis of the adhesion force of (a) pKO-αVβ1, (b) pKO-β1, or (c) pKO-αV fibroblasts to 2DX globular FN substrates, 2.5DX fibrillar FN matrices, and 3DX fibrillar FN matrices. Data for 2DX globular FN substrates and for 2.5DX fibrillar and 3DX fibrillar FN matrices is taken from SCFS experiments analyzed in Figure 2. The reference data for 2D globular FN substrates, 2.5D fibrillar FN matrices, and 3D fibrillar FN matrices is taken from Figure S2. Lines show linear fits of the data with shaded areas representing the 95% confidence interval. The slope of each linear fit is used to determine the adhesion strengthening of fibroblasts. Dots represent mean adhesion forces and error bars s.e.. *P* values were calculated using the two-tailed Mann-Whitney test.

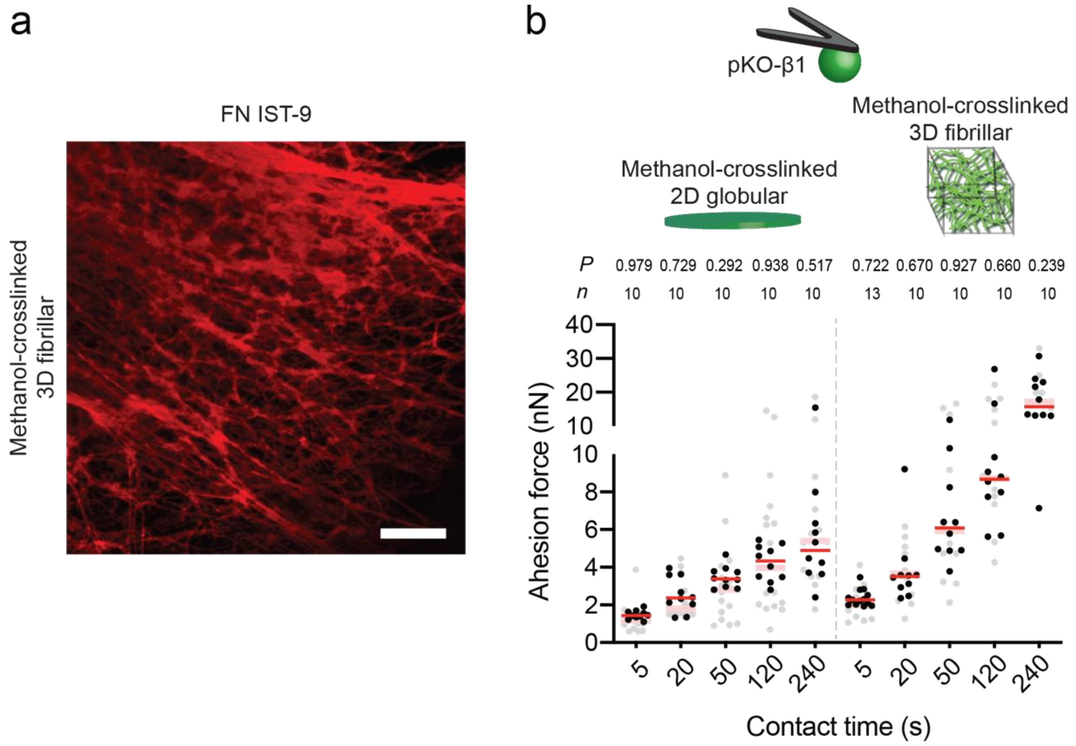

Figure S5. Adhesion initiation and strengthening of pKO-β1 fibroblasts to methanol-crosslinked 2D globular FN substrates and 3D fibrillar FN matrices. a) Representative fluorescence image of a methanol-crosslinked 3D fibrillar FN matrix. Unfolded FN is shown in red (FN IST-9 antibody). Scale bars, 20 µm. b) Adhesion forces of pKO-β1 fibroblasts to methanol-crosslinked 2D globular FN substrates and 3D fibrillar FN matrices. For reference, adhesion forces of fibroblasts to respective PFA-crosslinked FN substrates (taken from Figure 2f) are given in grey. *P* values compare between adhesion forces measured to methanol and PFA crosslinked FN substrates or matrices. Dots represent adhesion forces of single fibroblasts and red bars their median values. *n* indicates the number of fibroblasts tested. *P* values were calculated using the two-tailed Mann-Whitney test.

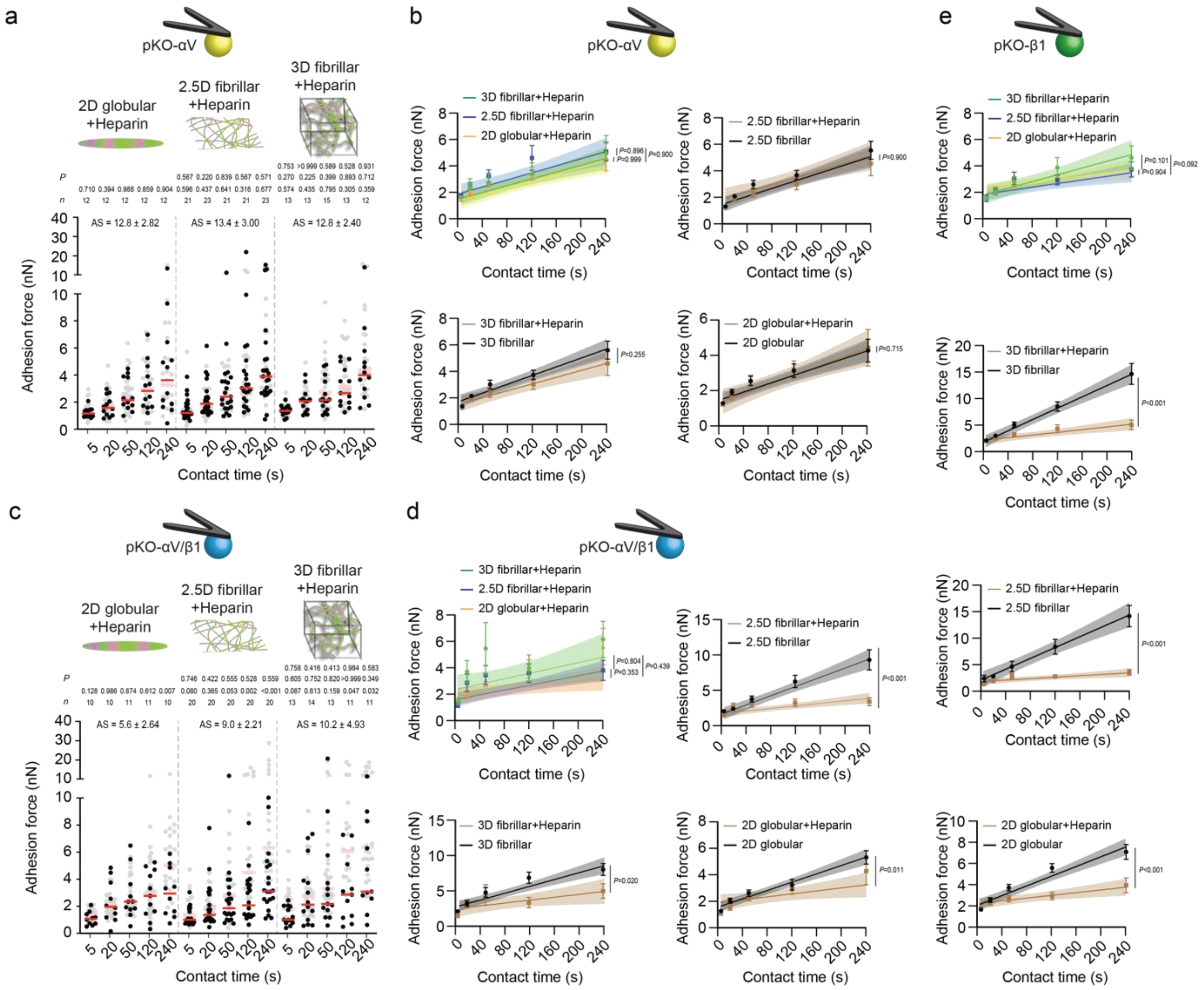

Figure S6. α5β1 integrin, but not αVβ3 integrin cooperate with syndecans to sense the fibrillarity of FN. (a) Adhesion forces and (b) adhesion strengthening of pKO-αV to 2D globular FN substrates, 2.5D fibrillar FN matrices, and 3D fibrillar FN matrices with heparin treatment. (c) Adhesion forces and (d) adhesion strengthening of pKO-αV/β1 to 2D globular, 2.5D fibrillar, and 3D fibrillar FN substrates with heparin treatment. e) Adhesion strengthening of pKO-β1 to heparin-treated substrates. Data is taken from experiments analyzed in Figure 3a. Reference data for 2D globular FN substrates, 2.5D fibrillar FN matrices, and 3D fibrillar FN matrices is taken from Figure S2, Supporting Information. In a and c, bottom *P* values compare without and with heparin treatment. Middle *P* values compare 2.5D fibrillar or 3D fibrillar matrices with 2D globular FN substrates. Top *P* values compare 2.5D fibrillar and 3D fibrillar FN matrices. Dots represent adhesion forces of single fibroblasts and red bars their median values. *n* indicates the number of fibroblasts tested. *P* values were calculated using two-tailed Mann-Whitney test. AS indicates the mean and s.e. of the adhesion strengthening rate (pN s^{-1}). In (b), (d) and (e), lines indicate the linear fit of the data and shaded areas the 95% confidence interval. Dots and bars represent mean adhesion force and s.e., respectively. *P* values were calculated using the two-tailed Mann-Whitney test.

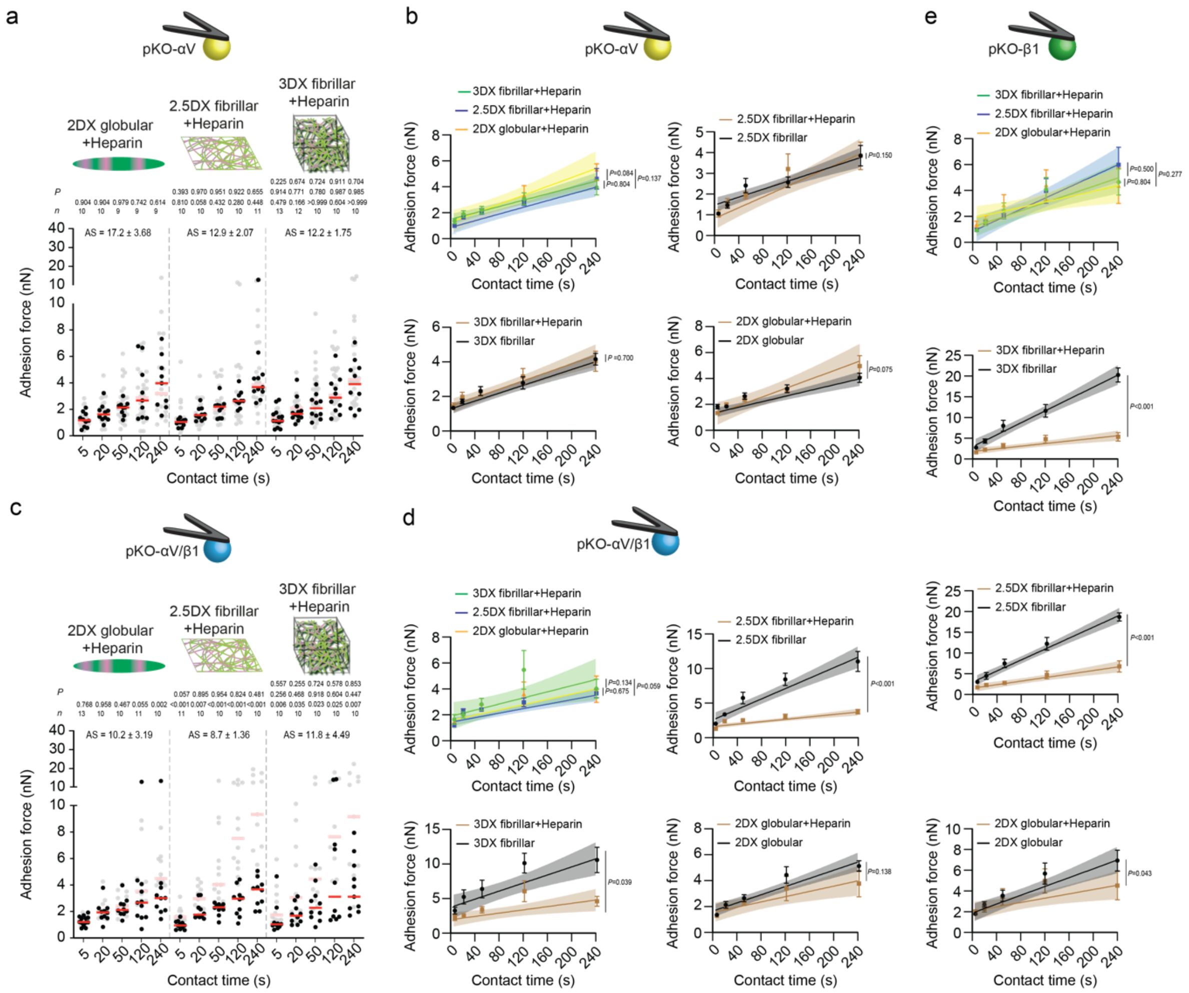

Figure S7. α5β1 integrin, but not αVβ3 integrin cooperate with syndecans to sense the stiffness of FN fibrils. a-b) Adhesion forces (a) and adhesion strengthening (b) of pKO-αV to crosslinked 2D (2DX) globular FN matrices, 2.5D (2.5DX) fibrillar FN matrices, and 3D (3DX) fibrillar FN matrices with heparin treatment. c-d) Adhesion forces (c) and adhesion strengthening (d) of pKO-αV/β1 fibroblasts to crosslinked 2D (2DX) globular FN substrates, 2.5D (2.5DX) fibrillar FN matrices, and 3D (3DX) fibrillar FN matrices with heparin treatment. e) Adhesion strengthening of pKO-β1 fibroblasts to crosslinked 2D (2DX) globular FN substrates, 2.5D (2.5DX) fibrillar FN matrices, and 3D (3DX) fibrillar FN matrices with heparin treatment. Data is taken from experiments analyzed in Figure 3b. Reference data for 2DX globular FN substrates, 2.5DX fibrillar FN matrices, and 3DX fibrillar FN matrices is taken from Figure S4, Supporting Information. In a and c, bottom *P* values compare without and with heparin treatment. Middle *P* values compare 2.5DX fibrillar FN matrices or 3DX fibrillar FN matrices with 2DX globular FN substrates. Top *P* values compare 2.5DX fibrillar and 3DX fibrillar FN matrices. Dots represent adhesion forces of single fibroblasts and red bars their median values. *n* indicates the number of fibroblasts tested. *P* values were calculated using two-tailed Mann-Whitney test. AS indicates the mean and s.e. of the adhesion strengthening rate (pN s^{-1}). In (b), (d) and (e), lines indicate the linear fit of the data and shaded areas the 95% confidence interval. Dots represent mean adhesion force and bars s.e.. *P* values were calculated using the two-tailed Mann-Whitney test.

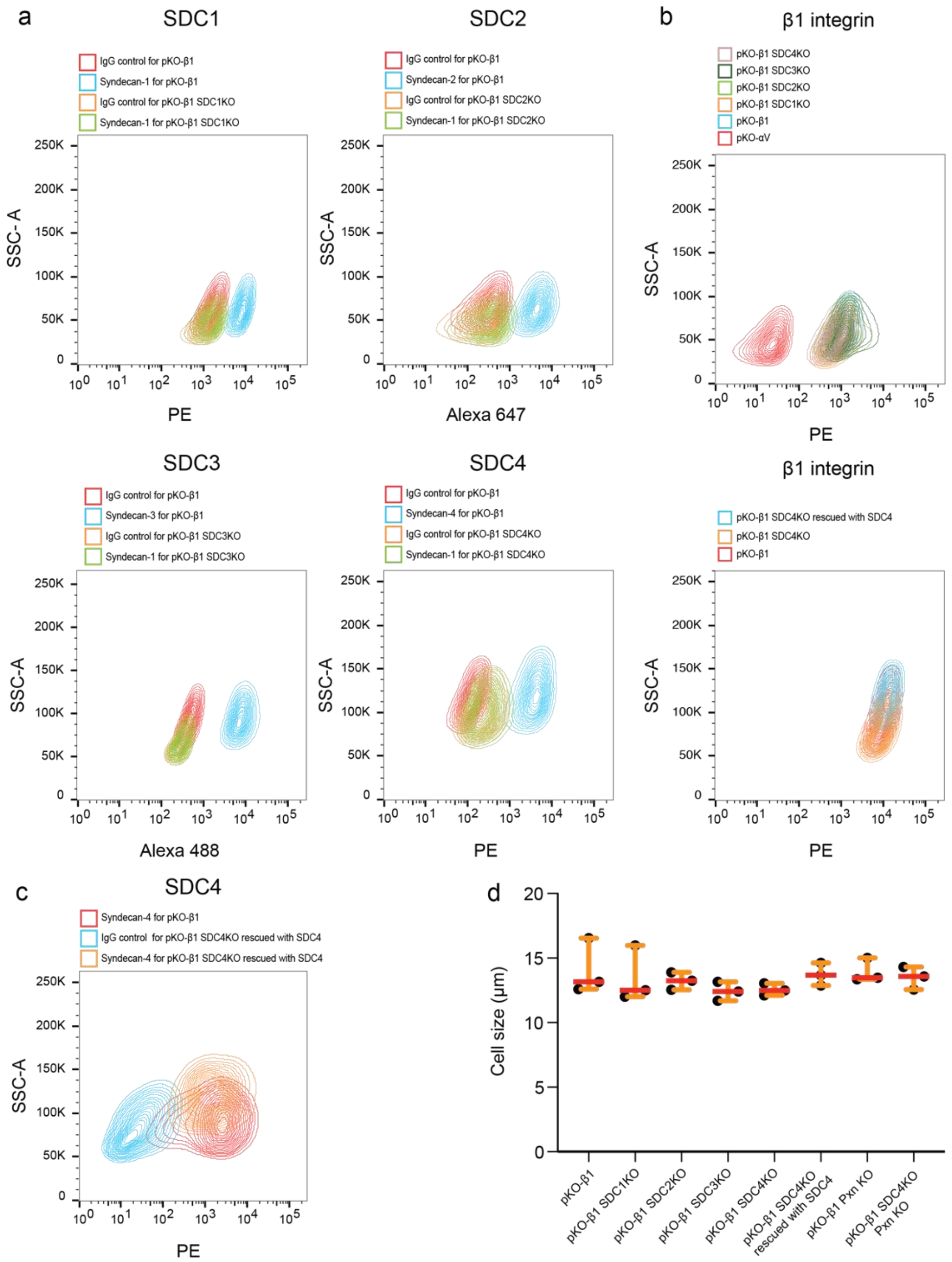

Figure S8. Syndecan and α5β1 integrin surface expression levels. a) Surface expression of syndecan-1 (SDC1) for pKO-β1 SDC1KO fibroblasts, syndecan-2 (SDC2 for pKO-β1 SDC2KO fibroblasts, syndecan-3 (SDC3) for pKO-β1 SDC3KO fibroblasts, or syndecan-4 (SDC4) for pKO-β1 SDC4KO fibroblasts as analyzed by flow cytometry to verify knockout of the target syndecans. pKO-β1 fibroblasts were used as a positive control. b) Surface expression of α5β1 integrin as analyzed for each syndecan knockout fibroblast line. pKO-β1 fibroblasts were used as a positive control, whereas pKO-αV fibroblasts were used as a negative control. Surface expression of α5β1 integrin as analyzed for pKO-β1 SDC4KO fibroblasts rescued with SDC4. pKO-β1 and pKO-β1 SDC4 KO fibroblasts were used as positive control. c) Surface expression of SDC4 for pKO-β1 SDC4KO fibroblasts rescued with SDC4 as analyzed by flow cytometry verifies the reintroduction of SDC4. pKO-β1 fibroblasts were used as a positive control. d) Cell size analysis of pKO-β1, pKO-β1 SDC1KO, pKO-β1 SDC2KO, pKO-β1 SDC3KO, pKO-β1 SDC4KO, pKO-β1 SDC4KO fibroblasts rescued with SDC4, pKO-β1 paxillin (Pxn) KO, and pKO-β1 SDC4KO Pxn KO fibroblasts. Dots represent the average cell diameter of each fibroblast batch tested. Red bars indicate the mean and orange bars the 95% confidential interval.

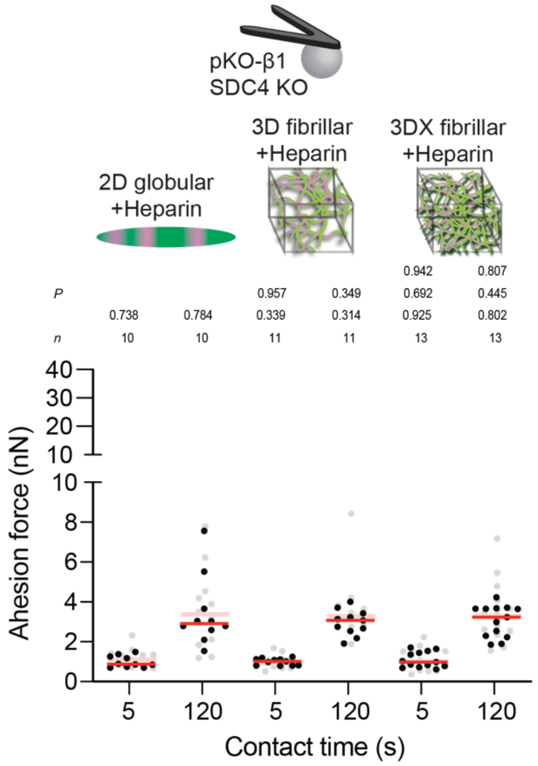

Figure S9. Heparin does not affect the adhesion of syndecan-4 knockout fibroblasts. Adhesion forces of pKO-β1 SDC4KO fibroblasts to heparin treated 2D globular FN substrates, 3D fibrillar FN matrices, and crosslinked 3D (3DX) fibrillar FN matrices at 5 s and 120 s contact times. Bottom *P* values compare without and with heparin treatment. Middle *P* values compare 3D fibrillar FN matrices or 3DX fibrillar FN matrices with 2D globular FN substrates. Top *P* values compare 3D fibrillar and 3DX fibrillar FN matrices. Dots represent adhesion forces of single fibroblasts and red bars their median values. *n* indicates the number of fibroblasts tested. *P* values were calculated using the two-tailed Mann-Whitney test.

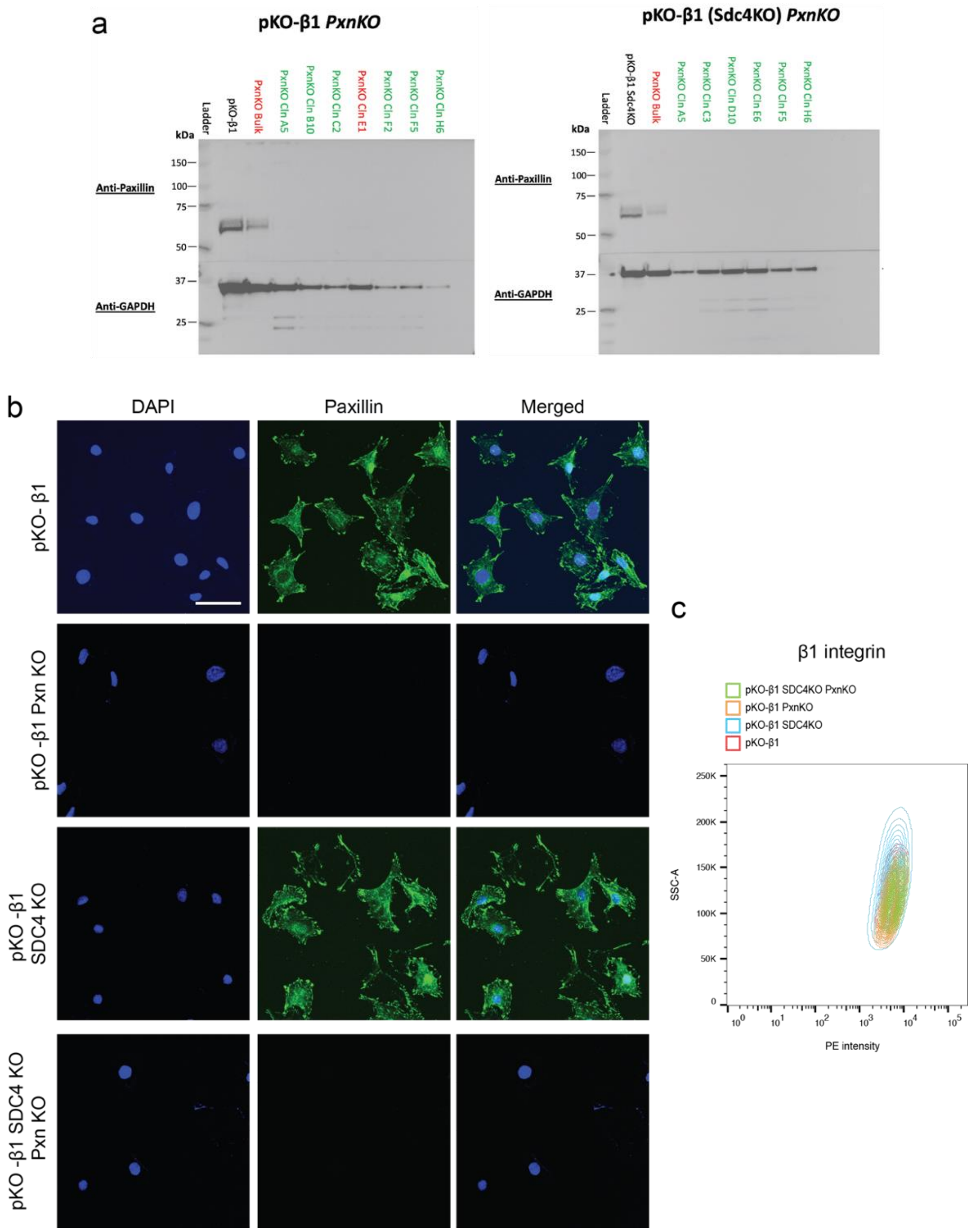

Figure S10. Paxillin knockout fibroblast production. a, b) Analyzing the expression of paxillin (Pxn) in pKO-β1, pKO-β1 SDC4 KO, pKO-β1 Pxn KO, and pKO-β1 SDC4 KO Pxn KO fibroblasts verifies the knockout of paxillin. pKO-β1 and pKO-β1 SDC4 KO fibroblasts were used as positive control. a) Western blot analysis of Pxn expression in pKO-β1 fibroblasts and different clones of pKO-β1 Pxn KO fibroblasts (left blot) as well as in pKO-β1 SDC4 KO fibroblasts and different clones of pKO-β1 SDC4 KO Pxn KO fibroblasts (right blot). Green written clones depict Pxn depletion. Among the clones, Cln A5, F5, and H6 for both pKO-β1 Pxn KO and pKO-β1 SDC4 KO Pxn KO fibroblasts were used in our studies. Pxn KO bulk stands for bulk sorted cells and Pxn KO Cln for single sorted clones. b) Fluoresence images of pKO-β1, pKO-β1 Pxn KO, pKO-β1 SDC4 KO, and pKO-β1 SDC4 KO Pxn KO fibroblasts stained against DAPI (for nuclei, blue) and paxillin (green). pKO-β1 and pKO-β1 SDC4 KO fibroblasts show positive paxillin signals, whereas pKO-β1 Pxn KO and pKO-β1 SDC4 KO Pxn KO fibroblasts do not show any paxillin signal. Scale bars, 50 µm. c) Surface expression of α5β1 integrin analyzed for pKO-β1 Pxn KO and pKO-β1 SDC4 KO fibroblasts. pKO-β1 and pKO-β1 SDC4 KO fibroblasts were used as positive control.

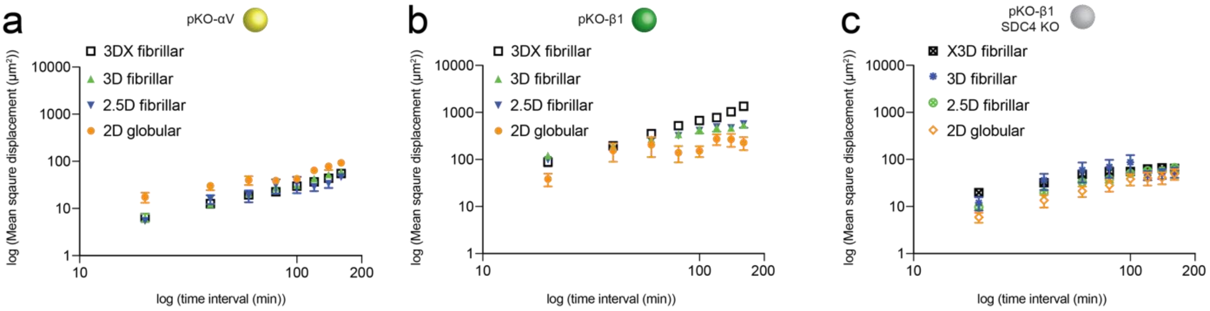

Figure S11. Fibroblast migration persistence analysis. Log-log plots with the log mean square displacement *versus* the log time interval for pKO-αV (a), pKO-β1 (b), and pKO-β1 SDC4 KO (c) fibroblasts used to calculate the migration persistence for Figure 5b, 5e, and 5h, respectively. Symbols and bars indicate the mean and s.e. of the mean square displacement values at the given time interval, respectively. *n* indicates the number of FN substrates tested (*n* = 10 for all substrates in a, *n* = 10 for 2D globular FN substrates, 12 for 2.5D fibrillar FN matrices, 14 for 3D fibrillar FN matrices, 15 for 3DX fibrillar FN matrices in b and 13 for 2D globular FN substrates, 13 for 2.5D fibrillar FN matrices, 11 for 3D fibrillar FN matrices, 12 for 3DX fibrillar FN matrices in c).

Statistical tests

pKO-αV

Day 0

	2.5D fibrillar	3D fibrillar	X3D fibrillar
2D globular	0.3808	0.6684	0.8384
2.5D fibrillar		0.7802	0.5381
3D fibrillar			0.6155

Day 3

	2.5D fibrillar	3D fibrillar	X3D fibrillar
2D globular	0.2230	0.1836	0.0853
2.5D fibrillar		0.9259	0.5407
3D fibrillar			0.4242

Day 7

	2.5D fibrillar	3D fibrillar	X3D fibrillar
2D globular	0.7818	0.7817	0.8690
2.5D fibrillar		0.8377	0.9842
3D fibrillar			0.8111

Day 14

	2.5D fibrillar	3D fibrillar	X3D fibrillar
2D globular	0.9263	0.4923	0.2556
2.5D fibrillar		0.6170	0.3622
3D fibrillar			0.7814

pKO-β1

Day 0

	2.5D fibrillar	3D fibrillar	X3D fibrillar
2D globular	0.1371	0.0593	0.1097
2.5D fibrillar		0.2874	0.8976
3D fibrillar			0.2541

Day 3

	2.5D fibrillar	3D fibrillar	X3D fibrillar
2D globular	<0.0001	0.0003	0.0003
2.5D fibrillar		0.4661	0.7237
3D fibrillar			0.5158

Day 7

	2.5D fibrillar	3D fibrillar	X3D fibrillar
2D globular	<0.0001	<0.0001	<0.0001
2.5D fibrillar		0.6706	0.7812
3D fibrillar			0.6982

Day 14

	2.5D fibrillar	3D fibrillar	X3D fibrillar
2D globular	<0.0001	<0.0001	<0.0001
2.5D fibrillar		0.0634	0.0548
3D fibrillar			0.1170

pKO-β1
SDC4 KO

Day 0

	2.5D fibrillar	3D fibrillar	X3D fibrillar
2D globular	0.8678	0.5376	0.6704
2.5D fibrillar		0.5640	0.8120
3D fibrillar			0.5647

Day 3

	2.5D fibrillar	3D fibrillar	X3D fibrillar
2D globular	0.8076	0.7811	0.8949
2.5D fibrillar		0.9873	0.7244
3D fibrillar			0.6978

Day 7

	2.5D fibrillar	3D fibrillar	X3D fibrillar
2D globular	0.5150	0.5376	0.8388
2.5D fibrillar		0.9704	0.5919
3D fibrillar			0.8676

Day 14

	2.5D fibrillar	3D fibrillar	X3D fibrillar
2D globular	0.8118	0.7559	0.9272
2.5D fibrillar		0.9860	0.9705
3D fibrillar			0.8107

Figure S12. Statistical analysis of the fibroblast proliferation data in Figure 6. *P* values lower than 0.05 (green) are considered significant.

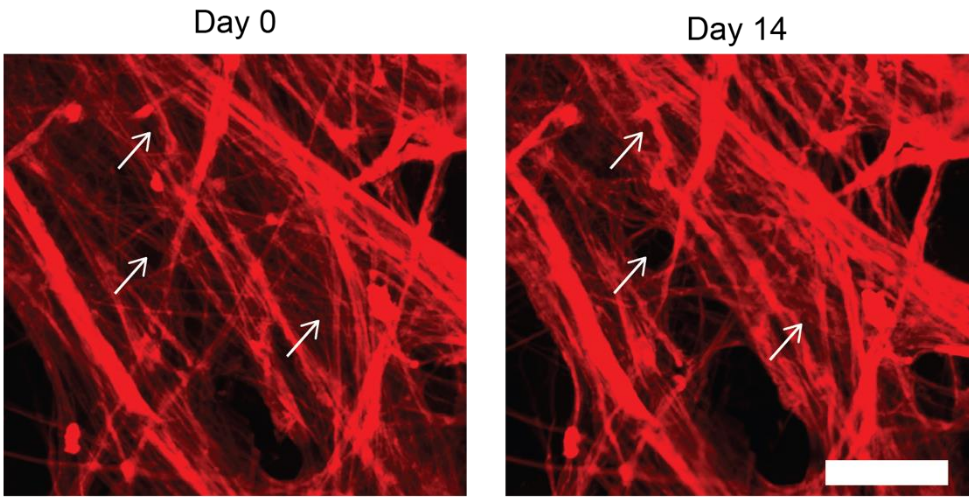

Figure S13. Remodeling of 3D fibrillar FN matrices after long-term fibroblast culture. Confocal images of 3D fibrillar FN matrices at day 0 and 14 after fibroblast culture. Before cell culture, FN was pre-stained with a dye (red, Alexa Fluor^{TM} 633 NHS (N-hydroxysuccinimide) ester). White arrows indicate areas where remodeling of fibrillar FN matrices can be observed. Scale bar, 100 µm.
